# Epidemiology, Clinical Features, and Outcomes of Multisystem Inflammatory Syndrome in Children (MIS-C) and Adolescents—a Live Systematic Review and Meta-analysis

**DOI:** 10.1007/s40124-022-00264-1

**Published:** 2022-05-06

**Authors:** Li Jiang, Kun Tang, Omar Irfan, Xuan Li, Enyao Zhang, Zulfiqar Bhutta

**Affiliations:** 1 Centre for Global Child Health, Hospital for Sick Children, Toronto, Canada; 2 Vanke School of Public Health, Tsinghua University, Beijing, China; 3 Department of Pharmacy Administration and Clinical Pharmacy, School of Pharmaceutical, Peking University, Beijing, China; 4 Institute for Global Health & Development, the Aga Khan University, Karachi, Pakistan

**Keywords:** COVID-19, Multisystem inflammatory syndrome, Children, Adolescents

## Abstract

**Purpose of Review:**

A multisystem inflammatory condition occurring in children and adolescents with COVID-19 has become increasingly recognized and widely studied globally. This review aims to investigate and synthesize evolving evidence on its clinical characteristics, management, and outcomes in pediatric patients.

**Recent Findings:**

We retrieved data from PubMed, EMBASE, Cochrane Library, WHO COVID-19 Database, Google Scholar, and preprint databases, covering a timeline from December 1, 2019, to July 31, 2021. A total of 123 eligible studies were included in the final descriptive and risk factor analyses. We comprehensively reviewed reported multisystem inflammatory syndrome in children (MIS-C) cases from published and preprint studies of various designs to provide an updated evidence on epidemiology, clinical, laboratory and imaging findings, management, and short-term outcomes. Latest evidence suggests that African black and non-Hispanic white are the two most common ethnic groups, constituting 24.89% (95% CI 23.30–26.48%) and 25.18% (95% CI 23.51–26.85%) of the MIS-C population, respectively. Typical symptoms of MIS-C include fever (90.85%, 95% CI 89.86–91.84%), not-specified gastrointestinal symptoms (51.98%, 95% CI 50.13–53.83%), rash (49.63%, 95% CI 47.80–51.47%), abdominal pain (48.97%, 95% CI 47.09–50.85%), conjunctivitis (46.93%, 95% CI 45.17–48.69%), vomiting (43.79%, 95% CI 41.90–45.68%), respiratory symptoms (41.75%, 95% CI 40.01–43.49%), and diarrhea (40.10%, 95% CI 38.23–41.97%). MIS-C patients are less likely to develop conjunctivitis (OR 0.27, 95% CI 0.11–0.67), cervical adenopathy (OR 0.21, 95% CI 0.07–0.68), and rash (OR 0.44, 95% CI 0.26–0.77), in comparison with Kawasaki disease patients. Our review revealed that the majority of MIS-C cases (95.21%) to be full recovered while only 2.41% died from this syndrome. We found significant disparity between low- and middle-income countries and high-income countries in terms of clinical outcomes.

**Summary:**

MIS-C, which appears to be linked to COVID-19, may cause severe inflammation in organs and tissues. Although there is emerging new evidence about the characteristics of this syndrome, its risk factors, and clinical prognosis, much remains unknown about the causality, the optimal prevention and treatment interventions, and long-term outcomes of the MIS-C patients.

**Supplementary Information:**

The online version contains supplementary material available at 10.1007/s40124-022-00264-1.

## Introduction

During the coronavirus disease 2019 (COVID-19) pandemic caused by severe acute respiratory syndrome coronavirus 2 (SARS-CoV-2), a multisystem inflammatory condition occurring in children and adolescents with COVID-19 has become increasingly recognized and widely studied globally. This newly emerged syndrome is referred to in different publications interchangeably as pediatric inflammatory multisystem syndrome temporally associated with SARS-CoV-2 (PIMS-TS) [1, 2] in Europe or multisystem inflammatory syndrome in children (MIS-C) associated with COVID-19 as per the US Center of Diseases and Control [3, 4]. Although children and adolescents make a small proportion of confirmed COVID-19 cases [5–8] and generally present with relatively mild symptoms or are asymptomatic [9], those with MIS-C can experience much severe symptoms and outcomes including inflammatory shock and multiorgan failure requiring intensive care and mechanical ventilation. Several National Health agencies and the World Health Organization (WHO) have released scientific briefs and advisories to recognize and manage this presentation of MIS-C related to COVID-19 [1, 3–5, 7, 10, 11].

According to our current understandings, MIS-C seems to develop in the post-infectious stage rather than during the acute infection stage of COVID-19 [12••] or to be more consistent with a subacute infection [13]. Its clinical characteristics are both similar and distinct from other well described inflammatory syndromes in children, including Kawasaki disease (KD) and toxic shock syndrome (TSS) [1, 11, 14••, 15, 16••, 17–23]. Since it was first reported back in April 2020, there have been accumulating evidences regarding the epidemiology, pathogenesis, clinical spectrum, and outcomes of MIS-C. Patients with MIS-C usually present with symptoms and laboratory findings related to the systemic hyperinflammation in addition to common COVID-19 symptoms and may develop myocarditis, cardiac dysfunction, acute kidney injury, and other organ damage as a result of the systemic hyperinflammatory response [12••]. However, there are still many clinical uncertainties about this new disease syndrome, including but not limited to its risk factors, varying symptoms, different clinical courses and disease severity, optimal management, and possible long-term outcomes.

In this systematic review, we comprehensively reviewed and synthesized currently available evidence on MIS-C to update the previous synthesis of a systemic review earlier in the pandemic [12••]. Furthermore, we aim to provide insights on identification and management of this syndrome in the clinical setting, its risk factors, and a unique comparison between higher-income and lower-income countries.

## Method

### Literature Search

We searched for observational studies on MIS-C to investigate and synthesize evolving evidence on its clinical characteristics, management, and outcomes. We retrieved data from PubMed, EMBASE, Cochrane Library, WHO COVID-19 Database, Google Scholar, and preprint databases, covering a timeline from December 1, 2019, to July 31, 2021. Complementary searches were conducted by manually searching the national public health websites and the John Hopkins Humanitarian Health Resource. The reference lists of all retrieved articles were examined as well. There was no language restriction applied for the search. The search terms applied and the specific search strategies for PubMed and other databases are provided in the Appendix Table 1. The search results from various databases were uploaded into Covidence Systematic Review Software (Veritas Health Innovation, 2016) for screening.

### Inclusion and Exclusion Criteria

We included observational studies on MIS-C cases and data related to MIS-C from national public health websites and official government reports. The studies included reported data on clinical characteristics, management, outcomes, and any effects of measures on risk factors on incidence of MIS-C and its severity. We excluded case reports, review articles, opinions, viewpoints, and modeling studies. Case reports were excluded to reduce any risk of over-representation of extreme cases. Studies with possible duplications of cases (e.g., overlapping time periods within the same institutions/cities/countries) were also excluded.

### Study Screening

Two review authors independently reviewed each title and abstract from the search results. Upon obtaining the full text, two reviewers independently screened the full text and decided whether to include or exclude the study, in accordance with the criteria specified previously. Any disagreements were resolved by an independent third author.

### Data Extraction

The data extracted from each study using standardized data abstraction forms included authors, country, study type, study period, study population and its description, demographic characteristics, SARS-CoV-2 RT-PCR or antibody test result, clinical symptoms, laboratory findings, imaging results, treatment plans, and outcomes. Signs and symptoms that occurred at any time during the patient’s clinical course were recorded. Only initial laboratory values were included in the analysis. For echocardiograms, we recorded any abnormal result, especially an abnormal ejection fraction, valvular dysfunction, any signs of pericarditis, coronary dilation, or aneurysm.

### Meta-analysis and Qualitative Synthesis

Continuous data were summarized as mean ± standard deviation. For each dichotomous outcome, the weighted mean proportion and 95% confidence interval (CI) were calculated. Logistic regression model was applied in identifying potential risk factors for MIS-C and severe outcomes related to MIS-C, and the fully adjusted, study-specific odds ratios (OR) for dichotomous data were combined to estimate the pooled OR with 95% CI. Pooled risk ratios (RR) with 95% CI between high-income countries (HICs) and low- and middle-income countries (LMICs) were also presented. The meta-analyses were performed using Stata 17 adopting the random-effects models. Statistical heterogeneity across studies was evaluated by calculating the *I*^2^ statistic. *I*^2^ values equal to or above 50% were considered as “significant” heterogeneity in this study. The characteristics, biases, and results of the included studies were summarized narratively. Studies not eligible for meta-analysis, due to a lack of sufficient data, were qualitatively synthesized. Analyses were completed initially by one author and subsequently reviewed by a second author.

### Assessment of Methodological Quality and Risk of Bias

Two independent reviewers assessed each included study for methodological quality. A quality assessment tool for observational studies was adopted from the National Heart, Lung, and Brain Institute (NHLBI) and Research Triangle Institute International [22]. Studies were not excluded based on study quality.

## Results

The systemic literature search yielded 1650 results during the search period. Of these, 135 studies were examined in full text and 123 were included in the final analysis (Appendix Fig. 1), with a total population size of 4475 children with MIS-C. The distribution of included studies according to country of origin and studies’ characteristics is summarized in Appendix Table 2 and Table 3. A total of 12 studies were excluded as they either presented overlapping data or did not provide age-disaggregated data for children or were commentaries, editorials, or reviews. A total of 98 articles were included for narrative review (32 studies) and descriptive meta-analysis (66 studies), and 37 studies were reviewed to derive any ratios for risk factors in the included cases.

**Fig. 1:**
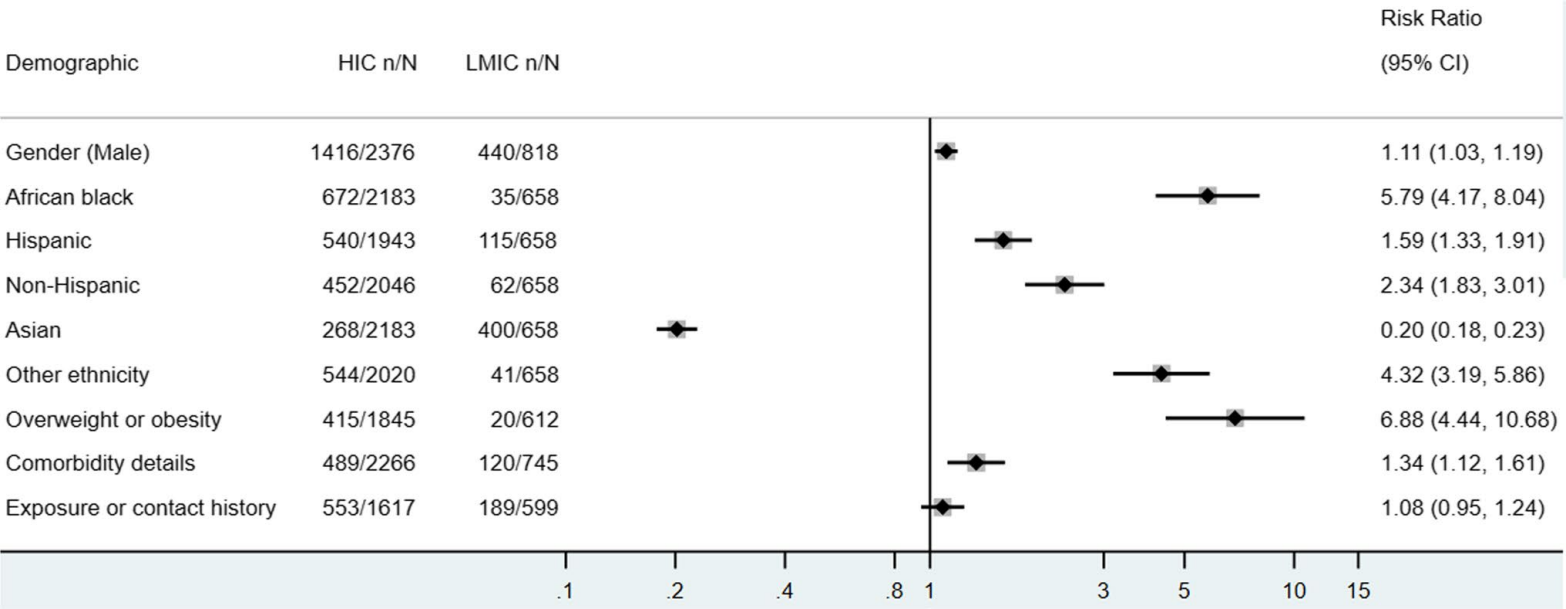
Demographics of included MIS-C cases in descriptive meta-analysis

### Demographic Characteristics of MIS-C Cases

The mean age of MIS-C patients was 8.1 ± 2.37 years, and 58.11% (1856/3194, 95% CI 56.40–59.82%) were boys. Demographics of included cases are as seen in Fig. 1. Among the 2841 individuals with race/ethnicity data, African black and Hispanic white are the two most common ethnic groups, constituting 24.89% (95% CI 23.30–26.48%) and 25.18% (95% CI 23.51–26.85%) of the MIS-C population, respectively, followed by Asian (23.51%, 95% CI 21.95–25.07%) and non-Hispanic white (19.01%, 95% CI 17.53–20.49%). Underlying comorbidities were reported in 20.23% (720/3625, 95% CI 18.79–21.66%) of the MIS-C patients, and 17.70% (525/3071, 95% CI 16.20–19.21%) are categorized as being overweight or obese. Approximately one-third (33.48%, 742/2216, 95% CI 31.52–35.45%) of the MIS-C cases had recent exposure or contact history with confirmed COVID-19 cases.

Few studies compared the characteristics of MIS-C with those of historical KD cases. Among the eight studies with the information of sex, there is no significant difference between MIS-C and KD regarding cases’ sex. There is a higher odd of African Black (OR 2.77, 95% CI 1.20–6.37) and a slightly lower odd of non-Hispanic White (OR 0.47, 95% CI 0.22–0.99) in the MIS-C population, while no significant difference in terms of the proportion of Hispanic Whites in the two populations were noted. When demographic data further disaggregated according to the country’s income level, MIS-C patients in HICs are associated with a higher probability of being male predisposed, African black, non-Hispanic white, and with underlying comorbidities (Appendix Table 4.1).

### Clinical Spectrum

**Fig. 2:**
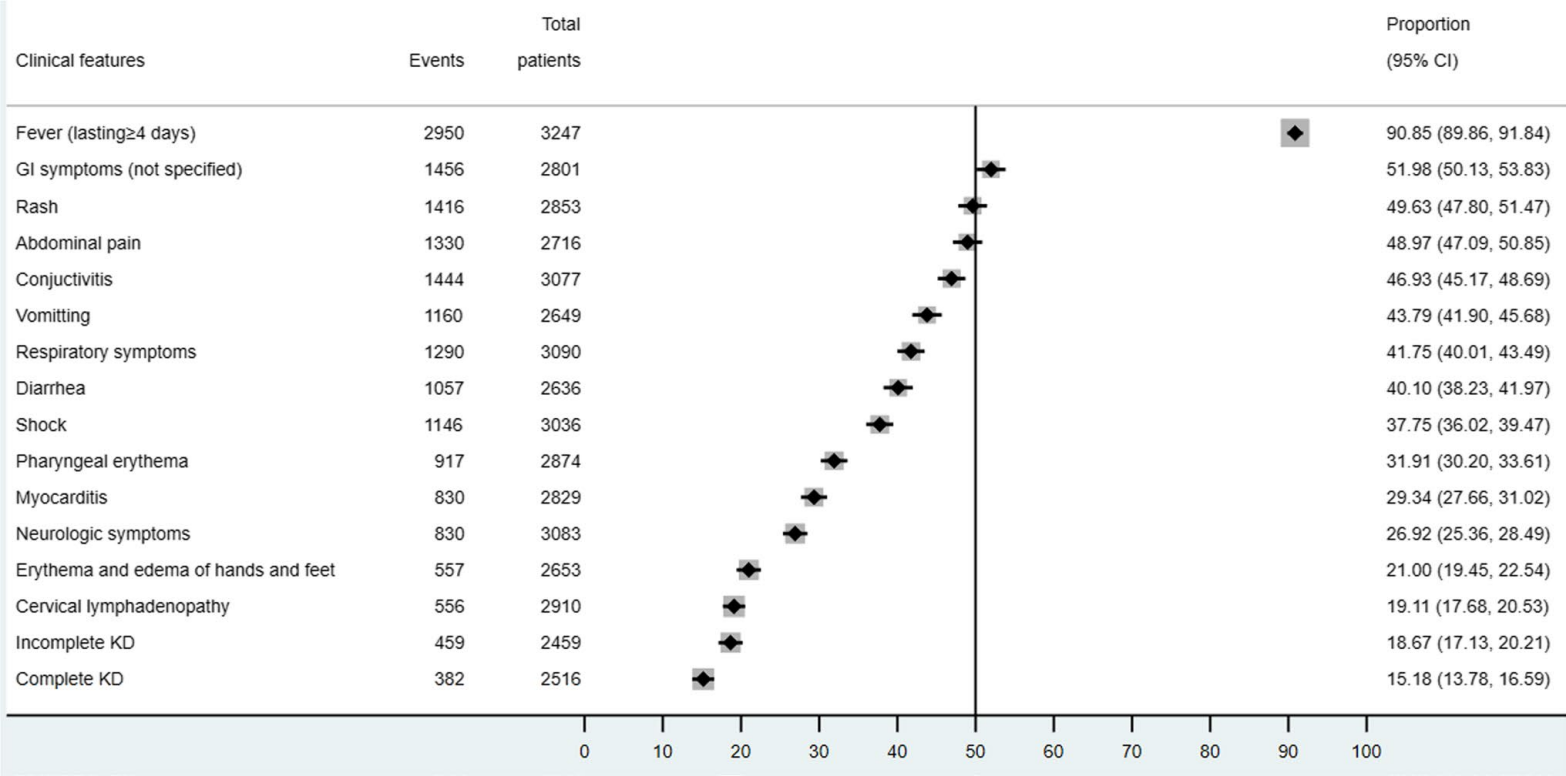
Clinical features of included MIS-C cases in descriptive meta-analysis

Prevalent clinical symptoms observed in MIS-C cases were fever (90.85%, 95% CI 89.86–91.84%), not-specified gastrointestinal symptoms (51.98%, 95% CI 50.13–53.83%), rash (49.63%, 95% CI 47.80–51.47%), abdominal pain (48.97%, 95% CI 47.09–50.85%), conjunctivitis (46.93%, 95% CI 45.17–48.69%), vomiting (43.79%, 95% CI 41.90–45.68%), respiratory symptoms (41.75%, 95% CI 40.01–43.49%), and diarrhea (40.10%, 95% CI 38.23–41.97%) as seen in Fig. 2. Less than one-third of patients developed pharyngeal erythema (31.91%, 95% CI 30.20–33.61%), myocarditis (29.34%, 95% CI 27.66–31.02%), neurologic symptoms (26.92%, 95% CI 25.36–28.49%), erythema and edema of hands and feet (21.00%, 95% CI 19.45–22.54%), and cervical lymphadenopathy (19.11%, 95% CI 17.68–20.53%). Only 18.67% (95% CI 17.13–20.21%) fulfilled the diagnostic criteria of incomplete KD and 15.18% (95% CI 13.78–16.59%) as complete KD. More than one-third of MIS-C patients presented with shock (37.75%, 95% CI 36.02–39.47%).

There are nine studies compared the clinical spectrum of MIS-C and KD. The occurrence of extremity changes, desquamation, lips and oral cavity changes, neurologic signs, arthritis and/or arthralgia, and coronary abnormalities did not differ between these two diseases. But MIS-C patients are less likely to develop the following symptoms comparing with KD patients: conjunctivitis (OR 0.27, 95% CI 0.11–0.67), cervical adenopathy (OR 0.21, 95% CI 0.07–0.68), and rash (OR 0.44, 95% CI 0.26–0.77). A small number of MIS-C cases met the criteria of complete KD (OR 0.24, 95% CI 0.17–0.35). On the other hand, compared to KD, it is more common to observe gastrointestinal symptoms (OR 11.44, 95% CI 5.18–25.26), mitral regurgitation (OR 6.62, 95% CI 1.81–24.20), pericardial effusion (OR 1.74, 95% CI 1.28–2.37), and pleural effusion (OR 19.21, 95% CI 3.10–119.16) in MIS-C cases.

**Fig. 3:**
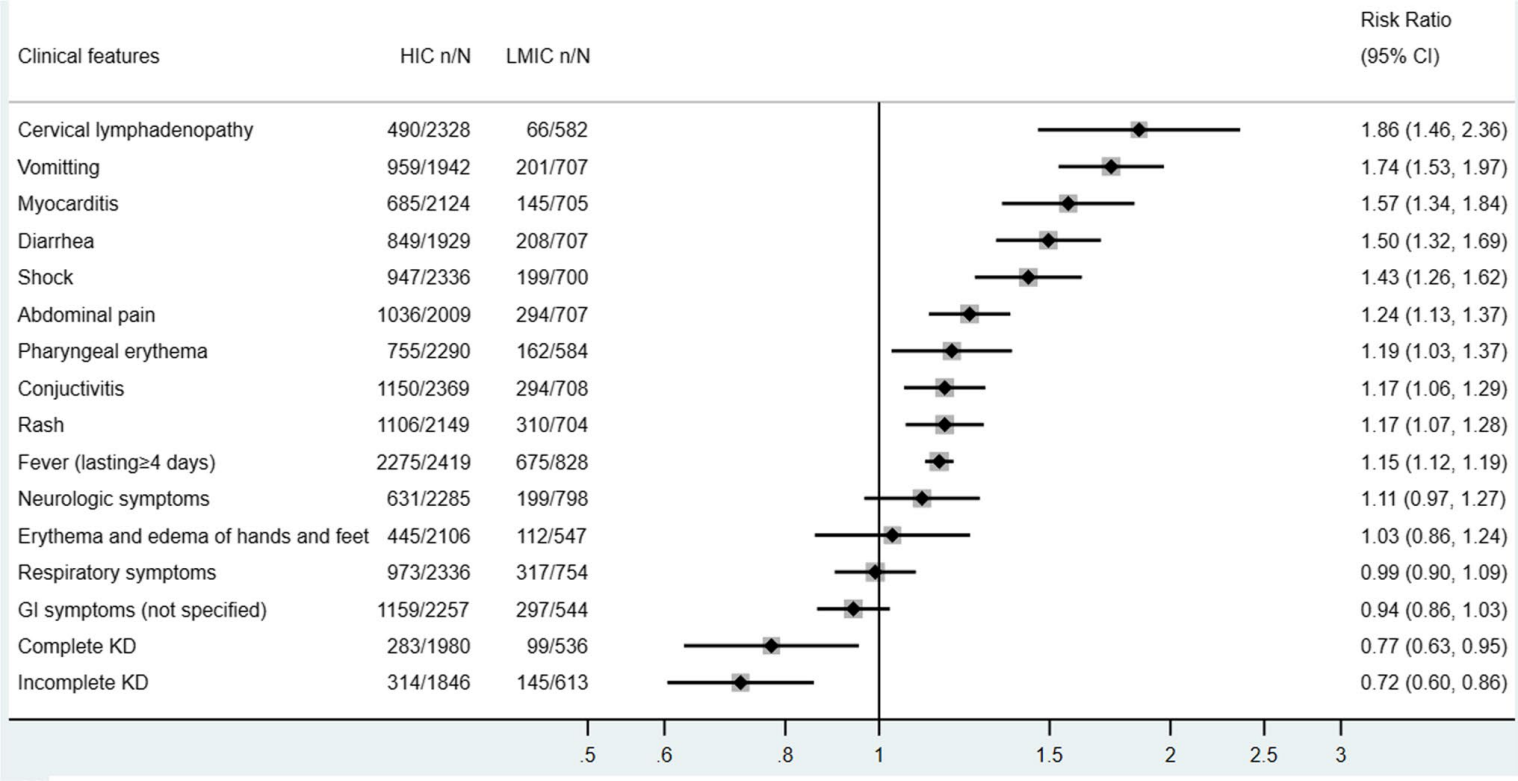
Clinical features of included HIC vs LMIC pediatric cases in descriptive meta-analysis

We compared the clinical presentations of MIS-C in HICs with those in LMICs (Fig. 3) and found that MIS-C patients in HICs are more likely to present with cervical lymphadenopathy, vomiting, myocarditis, diarrhea, abdominal pain, pharyngeal erythema, conjunctivitis, rash, and fever comparing to their counterparts in LMICs. They also have a higher risk of developing shock (*RR* = 1.43, 95% CI 1.26–1.62). On the other hand, MIS-C cases in HICs are at a significantly lower risk of fulfilling the diagnosis criteria of incomplete KD (*RR* = 0.72, 95% CI 0.60–0.86) and complete KD (*RR* = 0.77, 95% CI 0.63–0.95).

### Laboratory Findings

**Fig. 4:**
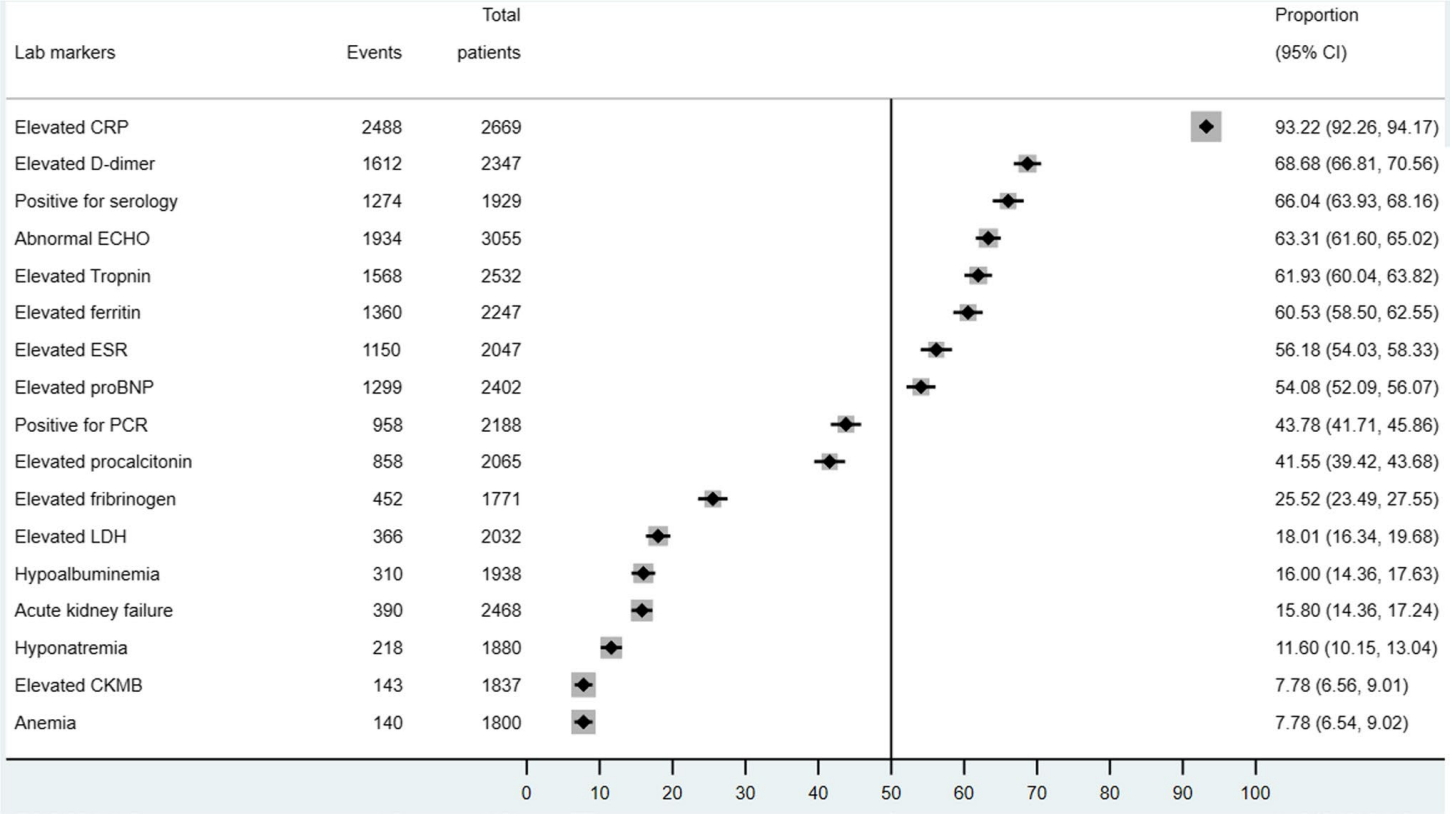
Laboratory findings of included pediatric cases in descriptive meta-analysis

**Fig. 5:**
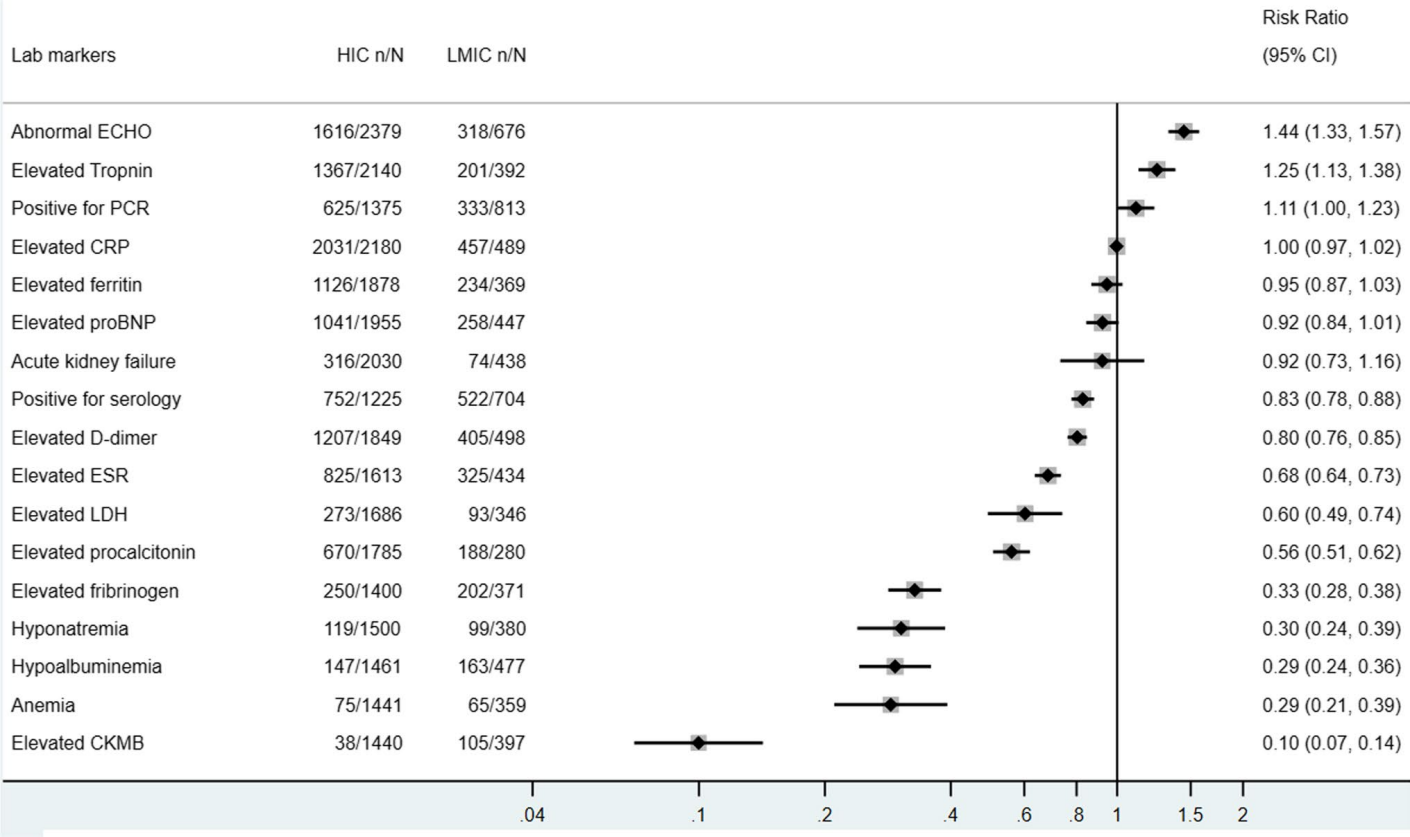
Laboratory findings of included HIC vs LMIC pediatric cases in descriptive meta-analysis

Of those reported COVID-19 testing (*n* = 2188), 39.0% reported PCR positivity and 66.7% reported positive antibodies. The majority of MIS-C patients presented with elevated inflammatory markers as seen in Fig. 4, including CRP (93.22%, 95% CI 93.26–94.17%), D-dimer (68.68%, 95% CI 66.81–70.56%), ferritin (60.53%, 95% CI 58.50–62.55%), ESR (56.18%, 95% CI 54.03–58.33%), and procalcitonin 41.55% (95% CI 39.42–43.68%), also had elevated procalcitonin. Other less common laboratory findings include acute kidney failure, hypoalbuminemia, hyponatremia, and anemia. There are obvious abnormalities in cardiac biomarkers and ultrasonographic findings in MIS-C patients. Around 61.93% (95% CI 60.04–63.82%) and 54.08% (95% CI 52.09–56.07%) had elevated troponin and pro-BNP, respectively, while 63.31% (95% CI 61.60–65.02%) presented with abnormal echocardiography findings. Most common echocardiographic abnormalities include left ventricular systolic dysfunction, coronary aneurysm, and dilatation. However, only 7.78% (95% CI 6.56–9.01%) of patients experienced an elevation in CKMB. A total of 66.04% (95% CI 63.93–68.16%) of the included MIS-C cases were positive for SARS-CoV-2 serology while less than half of these cases were positive for SARS-CoV-2 RT-PCR (43.78%, 95% CI 41.71–45.86%). Comparing with their counterparts in LMICs, MIS-C cases in HICs presented with lower or comparable risks in developing abnormal laboratory findings, but a higher risk of presenting with abnormal ECHO findings (RR 1.44, 95% CI 1.33–1.57) (Fig. 5).

### Risk Factors

Regarding the risk factors for developing MIS-C in pediatric COVID-19 population, twenty-eight studies compared the ethnicity of MIS-C patients and children with only COVID-19 and revealed that African Black have a lower risk of developing MIS-C after infecting SARS-CoV-2 (OR 0.63, 95% CI 0.49–0.82). Once infected, children with at least one comorbidity are more likely to develop MIS-C (OR 1.89, 95% CI 1.09–3.30), especially those with neuromuscular (OR 3.10, 95% CI 1.39–6.92) and respiratory diseases (OR 1.72, 95% CI 1.08–2.74) (Appendix Table 4.2).

And for the risk factors for admitting to ICU among MIS-C patients, we found that African Black patients with MIS-C (OR 1.50, 95% CI 1.15–1.95) and those with comorbidities (OR 1.36, 95% CI 1.04–1.78) had a higher risk of experiencing a more severe clinical course and being admitted to ICU (Appendix Table 4.3).

### Management and Outcomes

**Fig. 6:**
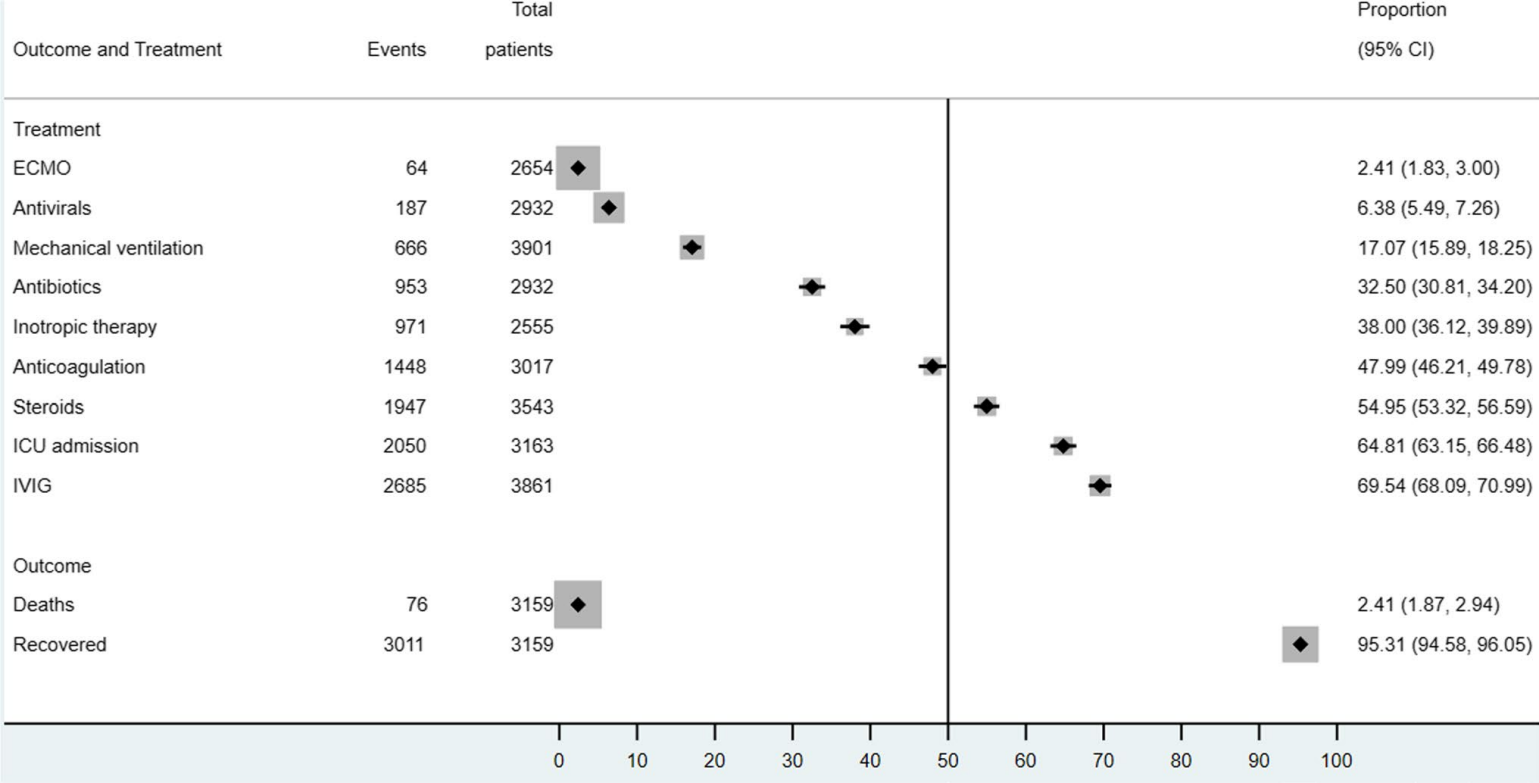
Management and outcomes of included pediatric cases in descriptive meta-analysis

From the evidence synthesis, the most common treatment modalities for MIS-C management protocols included intravenous immunoglobulin (69.54%, 95% CI 68.09–70.99%), steroids (54.95%, 95% CI 53.32–56.59%), anticoagulation (47.99%, 95% CI 46.21–49.78%), inotropic (38.00%, 95% CI 36.12–39.89%), and antibiotic therapy (32.50%, 95% CI 30.81–34.20%). Only 6.38% (95% CI 5.49–7.26%) had antiviral therapies. Although the ICU admission proportion of MIS-C is high (64.81%, 95% CI 63.15–66.48%), only 17.07% (95% CI 15.89–18.25%) of cases were put on mechanical ventilation and 2.41% (95% CI 1.83–3.00%) on extra-corporeal membrane oxygenation (ECMO). The vast majority of reported MIS-C patients (95.31%, 3613/3773, 95% CI 94.58–96.05%) recovered from the condition. Only 2.41% (76/3159, 95% CI 1.87–2.94%) of cases died (Fig. 6).

**Fig. 7:**
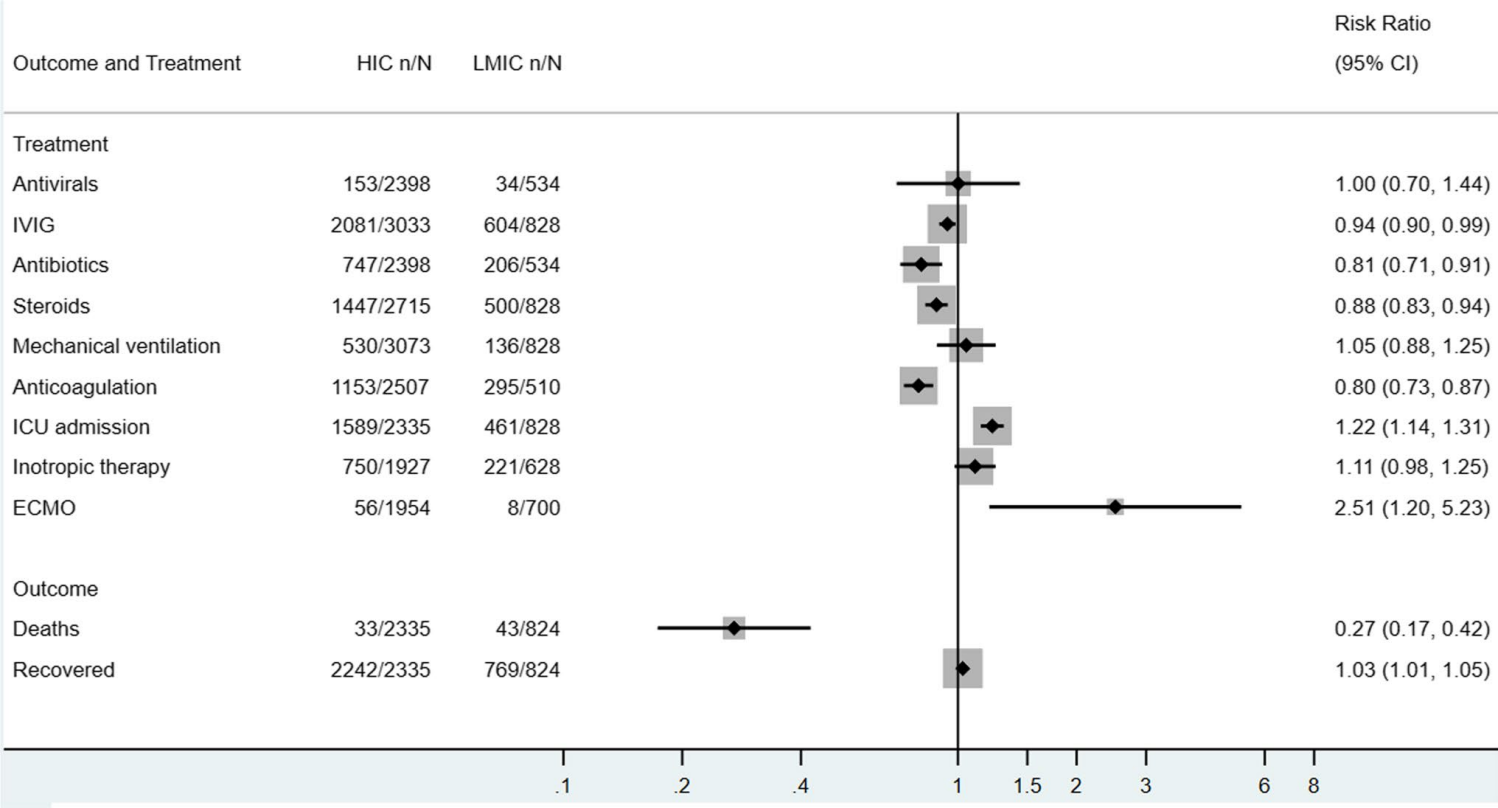
Management and outcomes of included HIC vs LMIC pediatric cases in descriptive meta-analysis

MIS-C patients in HICs were less likely to receive steroids (RR 0.88, 95% CI 0.83–0.94), antibiotics (RR 0.81, 95% CI 0.71–0.91), and anticoagulation therapy (RR 0.80, 95% CI 0.73–0.87). The proportion of recovered MIS-C cases did not differ significantly between HICs and LMICs. Despite a higher risk of ICU admission (RR 1.22, 95% CI 1.14–1.31) and ECMO application (RR 2.51, 95% CI 1.20–5.23) for MIS-C patients in HICs, they experienced a much lower risk of death compared to those diagnosed and treated in LMICs (*RR* = 0.27, 95% CI 0.17–0.42) (Fig. 7).

### Quality Assessment of Studies Included in the Meta-analysis

We performed quality assessment for the 66 studies included in the descriptive meta-analysis. Sixty-one of the included studies were considered of good quality and 5 of fair quality. Details of the quality assessment of included studies are presented in Appendix Table 5.

## Discussion

MIS-C is a novel disease associated with the infection of SARS-CoV-2 in children. It is characterized by the severe systemic inflammation and may lead to the damage and dysfunction of multiple organs, particularly the cardiac and coronary artery system. Children seem to develop symptoms and signs of MIS-C in the post-infection phase of COVID-19 instead of the acute infection phase and may progress into an advanced stage quickly [12••]. It is important to identify MIS-C and to initiate immunomodulatory therapies in a timely manner so as to help correct the hyperinflammatory state and prevent or minimize any target organ injuries. In this systematic review, we comprehensively reviewed reported MIS-C cases from published and preprint studies of various designs to provide an updated evidence on epidemiology, clinical spectrum, laboratory and imaging findings, management, and short-term outcomes. Our work will be able to give health care professionals and the general public a detailed picture about this newly emerged disease in this pandemic. Furthermore, we provide a unique comparison of reported MIS-C cases in HICs to LMICs to identify any regional differences regarding the clinical course of MIS-C.

### Risk Factors for MIS-C

Previous studies report certain groups to be at a higher risk of contracting the SARS-CoV-2 or developing a severe form of the infection. It has been reported that African Americans (different age groups, especially children) are at a higher risk of developing COVID-19 compared to other ethnicities [9, 24–26]. Other risk factors that have been reported to be associated with SARS-CoV-2 infection include the following: Hispanic ethnicity [25, 26], male gender [26], and living in a high-density community [26, 27]. Regarding the risk factors for developing MIS-C in the pediatric COVID-19 patients, current evidence is limited. We found in this systematic review that comorbidities in general are associated with a higher risk of MIS-C, while being African Black and obese/overweight reported a lower risk.

Our analysis revealed that children with at least one comorbidity have a higher risk of developing MIS-C once infected with SARS-CoV-2, particularly those with neuromuscular or respiratory diseases. However, in a prospective observational cohort study of 124 children [28] (63 confirmed or probable MIS-C cases and 61 cases with MIS-C-like presentations but with an alternative diagnosis), researchers found that only 35% of MIS-C cohort had underlying conditions. On the other hand, MIS-C cases included in our study have a mean age of 8.1 years with a slightly male predominance, which is consistent with the previous systematic reviews on MIS-C [29]. Nonetheless, neither age or sex could predict the occurrence of MIS-C once infected with SARS-CoV-2. Over 27% cases of the included MIS-C cases are African Black and Hispanic White each, followed by non-Hispanic White (21.27%). Although these groups take up a large proportion of MIS-C cases, which is consistent with previous studies [29–34], a comparable risk of developing MIS-C was observed. Instead, being African Black was associated with a lower risk for MIS-C presentation. This may be due to the disproportionately greater COVID-19 disease burden in these ethnic and racial groups [9, 24–26], which is resulting from complex factors, including nutrient deficiency, overcrowding living condition, and inadequate access to health care [26, 27].

Excessive production of inflammatory cytokines, as a direct reaction to the virus or as a postinfectious phenomenon, is associated with disease severity in adults with COVID-19 [35]. In adults, male sex, older age, obesity, diabetes, and certain pre-existing medical conditions have been identified as biological vulnerabilities for more severe COVID-19 outcomes [36–38]. In children and adolescents, age younger than 1 year of age, obesity, and pre-existing comorbidities including chronic cardiac and respiratory diseases are widely reported to be associated with more severe clinical outcomes [39, 40, 41••, 42]. Some studies also identified the presence of diarrhea [43] and significantly elevated CRP [40] as predictors of severe disease in children with COVID-19. Studies on factors predicting more severe outcomes in MIS-C patients are limited. Abrams et al. [44] identified that age older than 5 years was strongly associated with more severe outcomes in MIS-C. We identified only two studies comparing the risk of ICU admission due to MIS-C in various ethnic groups, four on comorbidities, and six on gender. In the pooled analysis, females, African American children, and those with any comorbidity are more likely to have a more severe clinical course and be admitted to ICU after developing MIS-C.

### Clinical Spectrum and Common Laboratory Findings

MIS-C was reported to have a very wide spectrum of presenting signs and symptoms, ranging from persistent fever and some characteristic features of Kawasaki disease to the most severe shock and multiorgan failures [12••]. Another systematic review conducted by Ahmed et al. found out that the most common symptoms of MIS-C are fever, gastrointestinal symptoms (abdominal pain, vomiting, and diarrhea), rash, and conjunctivitis [31]. Of 662 children, 60.1% included required vasopressor support and/or fluid resuscitation due to shock as a result of systemic inflammation and impaired cardiac output [31]. Previously published systematic reviews of MIS-C report higher prevalence of gastrointestinal symptoms in the majority of cases, rashes of varying descriptions in less than 50% of cases, and respiratory tract symptoms in a small proportion of cases [29–34, 45]. In this systematic review, we concluded the most prevalent symptoms of MIS-C include fever, gastrointestinal symptoms (especially abdominal pain, vomiting, and diarrhea), rash, and conjunctivitis. More than one-third of the cases included in our systematic review experienced shock (37.19%, 95% CI 35.40–38.98%) as a result of systemic inflammation and impaired cardiac output, and 63.91% of MIS-C cases were admitted into ICU.

While the most common clinical symptoms of COVID-19 in the pediatric population include fever, cough, nausea or vomiting, and diarrhea [9], there is overlap and distinctive features between MIS-C and pediatric COVID-19 regarding their clinical symptoms [29, 31].

The laboratory findings of MIS-C from our synthesis include significantly elevated inflammatory markers: CRP (92.59%), ferritin (60.53%), ESR (55.30%), and procalcitonin (39.05%). Over 50% MIS-C patients observed elevated cardiac markers consistent with previous reviews [29–34, 46, 47].

### MIS-C and Kawasaki Disease

MIS-C shares some similarities with KD, including male predominance, cases always presenting with persistent fever, shock, signs and symptoms of a systemic inflammatory response and multiorgan injuries, and their good response to steroids [12••]. Most notably, they can both lead to coronary artery aneurysm [12••, 48–52]. But there are differences between MIS-C and KD. MIS-C seems to impact an elder children population, more prevalent in African Black and Hispanic White instead of in the Asian group, and associated with greater elevation of inflammatory markers [14••, 17, 19, 22]. Our findings in this systematic review are consistent with previous literature. In the pooled analysis, children of African Black origin are more prevalent in MIS-C, different from KD, which is Asian-dominant. MIS-C cases are less commonly presented with some of the typical KD characteristic symptoms, such as rash, conjunctivitis, and cervical lymphadenopathy, and are more likely to present with pericardial, pleural effusion and mitral regurgitation in ECHO. Despite the overlapping symptoms of MIS-C and KD, less than one-fifth of MIS-C cases fulfill the diagnosis criteria of incomplete or complete KD (18.54%, 95% CI 16.94–20.14%, and 14.54%, 95% CI 13.10–15.97%, respectively).

### Current Treatment Plan and Outcomes for MIS-C

Some of the commonly employed treatment regimens for MIS-C include IVIG, steroid, antibiotics, anticoagulation, and inotropes. A national consensus from the UK [53••] suggests IVIG at a dose of 2 g/kg as the first-line therapy for children with PIMS-TS, which can be repeated to those not responding or partially responding to the first dose and intravenous methylprednisolone (10–30 mg/kg) as the second-line therapy. For patients who do not respond to the first- and second-line therapies, biological therapy can be commenced based on a multidisciplinary discussion and decision. They also recommended that intravenous antibiotics should be commenced in all patients, and antiviral therapy with remdesivir as the first choice might be considered for patients with positive SARS-CoV-2 RT-PCR or antigen testing result. Low-dose aspirin should be given and continued for a minimum of 6 weeks. We found in this systematic review that the management plan for MIS-C varies in different studies but was generally similar in HICs and LMICs.

If identified and managed timely and appropriately, MIS-C patients generally have a favorable outcome. Our review revealed that the majority of MIS-C cases (95.21%) fully recovered while only 2.41% died from this syndrome, consistent with other former systematic reviews [32–34, 45, 54]. There is paucity of reports on the long-term follow-up of MIS-C, possibly due to the fact that it is a newly emerged disease. A recent report [55••] on 45 children (< 21 years old) from New York, USA, who were followed at 1 to 4 weeks, 1 to 4 months, and 4 to 9 months post-discharge, was published. The median time of follow-up was around 6 months. The follow-up showed encouraging medium-term outcomes with 76% requiring intensive care but no mortalities, including rapid return to baseline of inflammatory markers and significant cardiac abnormalities in the majority of cases with immune derangements including lymphocytosis may extend up to several months after initial hospitalization. Continued caution and case reporting with follow-up are recommended to ensure timely management of children with MIS-C and to further inform pediatricians.

### Comparisons Between HICs and LMICs

In this systematic review, we further disaggregated our included patients according to the World Bank income levels and compared the epidemiology, clinical spectrum, treatment, and outcomes between the LMIC and HIC. Although with generally similar presentations and treatment plans, MIS-C cases in LMICs had a lower percentage of ICU admission and mechanical ventilation application but a higher risk of death. It may be explained by the relatively high disease burden and limited health care resources in LMICs.

### Strengths and Limitations

Our review was conducted in a systematic manner and comprehensively included and analyzed current available data on MIS-C. It provided an overview of the epidemiology, clinical spectrum, management, and short-term outcomes of this newly emerged disease so as to shed some light on current diagnosis, management, and future researches of MIS-C. But our review also has some limitations. Firstly, although the quality of the majority of included studies was shown to be fair or good, a lot of included studies were case reports/series, limiting its methodological quality. Secondly, original studies investigating the risk factors for developing MIS-C and leading to a more severe clinical course of MIS-C are limited. Furthermore, many children with COVID-19 are asymptomatic and COVID-19 cases of certain demographic and socioeconomic groups may be underreported. It also makes it difficult to identify and confirm the risk factors for developing MIS-C. Thirdly, case definition in different studies may vary, particularly at the early stage of the pandemic. Together with various study design, it resulted in the great heterogeneity of our included studies.

### Future Research Directions

The pathogenesis and risk factors of MIS-C are still unclear. And it remains challenging to make an early identification and a precise diagnosis for MIS-C in the clinical setting due to its wide clinical spectrum and great overlap with other infectious disease and autoimmune disorders in clinical presentations. Researches focusing on these areas are crucial in guiding either public health policy making or clinical diagnosis and management of MIS-C.

As a newly emerged disease, we have very limited evidence on the long-term outcome of MIS-C. Particularly, cardiac abnormalities were not only restricted to critically ill children ^28^, making it necessary to follow up all patients with MIS-C with and without immediately apparent cardiac complications for long-term cardiac outcomes.

Furthermore, greater efforts should be made to improve the health equity of MIS-C patients in LMICs or from certain vulnerable population groups. For example, most of the immunomodulator medications advised to treat MIS-C are either unavailable or unaffordable in LMICs. Although the immunosuppressive steroids are affordable and accessible, their safeness, appropriate type, dose, route, and duration of use should be carefully studied in the LMICs context given the high prevalence and limited diagnostic capacity to exclude tuberculosis and HIV infections there [34].

## Conclusion

MIS-C is a novel disease associated with the infection of SARS-CoV-2 in children. It is characterized by the severe systemic inflammation and may lead to the damage and dysfunction of multiple organs, particularly the cardiac and coronary artery system. We provide an updated group of evidence on epidemiology, clinical spectrum, laboratory and imaging findings, management, and short-term outcomes. Current evidence suggests that comorbidities may be associated with a higher risk of MIS-C, while being African Black and obese/overweight saw a lower risk. MIS-C cases in LMICs had a lower percentage of ICU admission and mechanical ventilation application but a higher risk of death. More evidence is needed for the causality, the optimal prevention and treatment interventions, and long-term outcomes of the MIS-C patients.

## Supplementary Information

Below is the link to the electronic supplementary material.

Supplementary file1 (DOCX 524 KB)
