# Supplementary material for: Epidemiology, Clinical Features, and Outcomes of Multisystem Inflammatory Syndrome in Children (MIS-C) and Adolescents—a Live Systematic Review and Meta-analysis
Source: Curr Pediatr Rep. 2022 May 6;10(2):19–30. doi: 10.1007/s40124-022-00264-1 (PMC9072767; doi:10.1007/s40124-022-00264-1)

**Appendices**

**Table 1: Search Strategy for MEDLINE**

**Table 2: Distribution of studies (N=98) according to Country of origin**

**Table 3: Characteristics of studies included in the descriptive meta-analysis (N=98)**

**Table 4.1: Comparison between MIS-C and KD**

**Table 4.2: The risk factors for developing MIS-C in pediatric COVID-19 population**

**Table 4.3: The risk factors of ICU admission among MIS-C patients**

**Table 5a: Quality assessment of studies included in the descriptive meta-analysis (N=66)**

**Table 5b: Quality assessment of studies included in the risk factor meta-analysis (N=37)**

**Figure 1: Flow diagram of the systematic review process**

**Figure 2: Global map of distribution of the included MIS-C cases (N=3817) in the studies included in the descriptive meta-analysis**

**Figure 3. Comparison of Demographics of included HIC vs LMIC pediatric cases in descriptive meta-analysis**

**Figure 4. Outcomes of included HIC vs LMIC pediatric cases in descriptive meta-analysis**

**Table 1: Search Strategy for MEDLINE**

(((coronavirus or "covid 2019" or "SARS2" or "SARS" or "SARS CoV19" or "severe acute respiratory syndrome coronavirus 2" or "coronavirus infection" or "severe acute respiratory pneumonia outbreak" or "novel cov" or 2019ncov or sars cov2 or cov2 or ncov or covid or covid19 or coronaviridae or "corona virus")) OR (coronavirus[MeSH Terms]) ) AND ("multisystem inflammatory syndrome" or MISC or MIS-C or PIMTS or "Kawasaki-like disease" or kawasaki or "macrophage activation syndrome" or Multisystem or "COVID-19-associated multisystem inflammatory syndrome")

**Table 2: Distribution of studies (N=67) according to Country of origin included in the descriptive meta-analysis**

| **Country** | **Count of reports** | **Sum of COVID-19 cases** |
| --- | --- | --- |
| Turkey | 9 | 257 |
| Iran | 2 | 55 |
| India | 7 | 144 |
| South Africa | 1 | 23 |
| Brazil | 2 | 67 |
| Pakistan | 3 | 48 |
| Tunisia | 1 | 8 |
| Serbia | 1 | 3 |
| UK | 9 | 351 |
| Italy | 3 | 12 |
| France | 7 | 118 |
| Spain | 5 | 171 |
| Poland | 1 | 39 |
| Chile | 2 | 53 |
| Saudi Arabia | 1 | 10 |
| Switzerland | 2 | 10 |
| South Korea | 1 | 3 |
| Germany | 1 | 9 |
| USA | 2* | 1134 |
| Qatar | 1 | 7 |
| Mutli-centric studies | 6 | 1311 |
| **Total** | **67** | **3871** |

**31 studies from the US are already included in Abrams et al as observed through overlapping timelines and institutions.*

**Table 3: Characteristics of included studies (N=106)**

| Study and year | Country | World Bank Country Classification  LMIC: Low and middle-income Country, HIC: High-income Country | Total number (N) | Mean age (years) | Males (N) | Past medical history and Contact history | Presenting signs and symptoms | Covid19 testing and radiological imaging | | Management (other than supportive care) and Outcomes |
| --- | --- | --- | --- | --- | --- | --- | --- | --- | --- | --- |
| Aydin F 2021 | Turkey | LMIC | 26 |  | 16 | Contact history (n=NR)  Comorbidities (n=NR) | Fever (n=26), Conjunctivitis (n=15), Erythema and cracking of lips, tongue, oral mucosa (n=NR), Cervical lymphadenopathy (n=NR), Erythema and edema of hands and feet (n=NR), Rash (n=15), Complete KD (n=NR), Incomplete KD (n=NR), Myocarditis (n=NR), Shock (n=NR), Diarrhea (n=NR), Vomiting (n=NR), GI symptoms (n=26), Neurological symptoms (n=NR) | | PCR positivity (n=26), Serology positivity (n=26), Abnormal echocardiography (n=NR) | Steroids (n=25), IVIG (n=26), anticoagulants (n=NR), inotropes (n=NR), antivirals (n=NR), Mechanical ventilation (n=4)  Recovered (n=25)  ICU admission (n=26), Deaths (n=1), |
| Akca K 2021 | Turkey | LMIC | 4 |  | 1 | Contact history (n=0)  Comorbidities (n=NR) | Fever (n=4), Conjunctivitis (n=2), Erythema and cracking of lips, tongue, oral mucosa (n=2), Cervical lymphadenopathy (n=4), Erythema and edema of hands and feet (n=3), Rash (n=2), Complete KD (n=0), Incomplete KD (n=3), Myocarditis (n=1), Shock (n=0), Diarrhea (n=0), Vomiting (n=1), GI symptoms (n=NR), Neurological symptoms (n=NR) | | PCR positivity (n=1), Serology positivity (n=2), Abnormal echocardiography (n=1) | Steroids (n=3), IVIG (n=4), anticoagulants (n=2), inotropes (n=1), antivirals (n=1), Mechanical ventilation (n=1)  Recovered (n=3)  ICU admission (n=1), Deaths (n=1), |
| Antunez Montez 2021 | 5 Latin American countries | LMIC | 95 |  | 25 | Contact history (n=NR)  Comorbidities (n=11) | Fever (n=55), Conjunctivitis (n=NR), Erythema and cracking of lips, tongue, oral mucosa (n=NR), Cervical lymphadenopathy (n=NR), Erythema and edema of hands and feet (n=NR), Rash (n=NR), Complete KD (n=NR), Incomplete KD (n=NR), Myocarditis (n=2), Shock (n=14), Diarrhea (n=NR), Vomiting (n=NR), GI symptoms (n=43), Neurological symptoms (n=12) | | PCR positivity (n=95), Serology positivity (n=72), Abnormal echocardiography (n=9) | Steroids (n=27), IVIG (n=38), anticoagulants (n=NR), inotropes (n=14), antivirals (n=NR), Mechanical ventilation (n=9)  Recovered (n=93)  ICU admission (n=20), Deaths (n=2), |
| De Farias 2021 | Brazil | LMIC | 11 |  | 9 | Contact history (n=8)  Comorbidities (n=8) | Fever (n=11), Conjunctivitis (n=5), Erythema and cracking of lips, tongue, oral mucosa (n=0), Cervical lymphadenopathy (n=0), Erythema and edema of hands and feet (n=0), Rash (n=5), Complete KD (n=4), Incomplete KD (n=1), Myocarditis (n=0), Shock (n=5), Diarrhea (n=0), Vomiting (n=0), GI symptoms (n=0), Neurological symptoms (n=0) | | PCR positivity (n=2), Serology positivity (n=9), Abnormal echocardiography (n=7) | Steroids (n=11), IVIG (n=10), anticoagulants (n=9), inotropes (n=7), antivirals (n=NR), Mechanical ventilation (n=7)  Recovered (n=11)  ICU admission (n=11), Deaths (n=2), |
| Mamisha 2021 | Iran | LMIC | 45 |  | 24 | Contact history (n=14)  Comorbidities (n=6) | Fever (n=41), Conjunctivitis (n=23), Erythema and cracking of lips, tongue, oral mucosa (n=0), Cervical lymphadenopathy (n=9), Erythema and edema of hands and feet (n=18), Rash (n=24), Complete KD (n=0), Incomplete KD (n=31), Myocarditis (n=8), Shock (n=2), Diarrhea (n=16), Vomiting (n=23), GI symptoms (n=0), Neurological symptoms (n=0) | | PCR positivity (n=10), Serology positivity (n=35), Abnormal echocardiography (n=17) | Steroids (n=27), IVIG (n=18), anticoagulants (n=NR), inotropes (n=NR), antivirals (n=NR), Mechanical ventilation (n=0)  Recovered (n=40)  ICU admission (n=5), Deaths (n=5), |
| Jain 2021 | India | LMIC | 23 |  | 11 | Contact history (n=8)  Comorbidities (n=NR) | Fever (n=23), Conjunctivitis (n=11), Erythema and cracking of lips, tongue, oral mucosa (n=4), Cervical lymphadenopathy (n=NR), Erythema and edema of hands and feet (n=3), Rash (n=14), Complete KD (n=NR), Incomplete KD (n=NR), Myocarditis (n=15), Shock (n=15), Diarrhea (n=15), Vomiting (n=15), GI symptoms (n=NR), Neurological symptoms (n=0) | | PCR positivity (n=9), Serology positivity (n=7), Abnormal echocardiography (n=NR) | Steroids (n=22), IVIG (n=15), anticoagulants (n=NR), inotropes (n=NR), antivirals (n=3), Mechanical ventilation (n=9)  Recovered (n=22)  ICU admission (n=9), Deaths (n=1), |
| Webb 2021 | South Africa | LMIC | 23 |  | 6 | Contact history (n=3)  Comorbidities (n=8) | Fever (n=23), Conjunctivitis (n=15), Erythema and cracking of lips, tongue, oral mucosa (n=NR), Cervical lymphadenopathy (n=NR), Erythema and edema of hands and feet (n=NR), Rash (n=20), Complete KD (n=7), Incomplete KD (n=9), Myocarditis (n=21), Shock (n=13), Diarrhea (n=13), Vomiting (n=0), GI symptoms (n=NR), Neurological symptoms (n=5) | | PCR positivity (n=4), Serology positivity (n=NR), Abnormal echocardiography (n=NR) | Steroids (n=15), IVIG (n=23), anticoagulants (n=NR), inotropes (n=9), antivirals (n=NR), Mechanical ventilation (n=6)  Recovered (n=15)  ICU admission (n=12), Deaths (n=0), |
| Shahbaznejad 2021 | Iran | LMIC | 10 |  | 6 | Contact history (n=10)  Comorbidities (n=2) | Fever (n=10), Conjunctivitis (n=3), Erythema and cracking of lips, tongue, oral mucosa (n=4), Cervical lymphadenopathy (n=0), Erythema and edema of hands and feet (n=6), Rash (n=8), Complete KD (n=1), Incomplete KD (n=1), Myocarditis (n=1), Shock (n=2), Diarrhea (n=7), Vomiting (n=6), GI symptoms (n=9), Neurological symptoms (n=1) | | PCR positivity (n=4), Serology positivity (n=2), Abnormal echocardiography (n=3) | Steroids (n=4), IVIG (n=9), anticoagulants (n=3), inotropes (n=4), antivirals (n=10), Mechanical ventilation (n=1)  Recovered (n=9)  ICU admission (n=9), Deaths (n=1), |
| Lima setta 2021 | Brazil | LMIC | 56 |  | 39 | Contact history (n=27)  Comorbidities (n=11) | Fever (n=56), Conjunctivitis (n=26), Erythema and cracking of lips, tongue, oral mucosa (n=17), Cervical lymphadenopathy (n=15), Erythema and edema of hands and feet (n=NR), Rash (n=38), Complete KD (n=26), Incomplete KD (n=16), Myocarditis (n=10), Shock (n=33), Diarrhea (n=30), Vomiting (n=21), GI symptoms (n=NR), Neurological symptoms (n=27) | | PCR positivity (n=14), Serology positivity (n=19), Abnormal echocardiography (n=NR) | Steroids (n=31), IVIG (n=50), anticoagulants (n=29), inotropes (n=NR), antivirals (n=5), Mechanical ventilation (n=6)  Recovered (n=53)  ICU admission (n=56), Deaths (n=1), |
| Dhanalakshmi 2021 | India | LMIC | 19 |  | 8 | Contact history (n=0)  Comorbidities (n=1) | Fever (n=19), Conjunctivitis (n=9), Erythema and cracking of lips, tongue, oral mucosa (n=9), Cervical lymphadenopathy (n=6), Erythema and edema of hands and feet (n=10), Rash (n=12), Complete KD (n=7), Incomplete KD (n=0), Myocarditis (n=0), Shock (n=10), Diarrhea (n=3), Vomiting (n=6), GI symptoms (n=0), Neurological symptoms (n=6) | | PCR positivity (n=4), Serology positivity (n=8), Abnormal echocardiography (n=3) | Steroids (n=11), IVIG (n=15), anticoagulants (n=16), inotropes (n=6), antivirals (n=1), Mechanical ventilation (n=0)  Recovered (n=19)  ICU admission (n=12), Deaths (n=0), |
| Falah 2021 | Pakistan | LMIC | 10 |  | 8 | Contact history (n=2)  Comorbidities (n=0) | Fever (n=10), Conjunctivitis (n=9), Erythema and cracking of lips, tongue, oral mucosa (n=9), Cervical lymphadenopathy (n=3), Erythema and edema of hands and feet (n=5), Rash (n=8), Complete KD (n=5), Incomplete KD (n=5), Myocarditis (n=3), Shock (n=6), Diarrhea (n=3), Vomiting (n=0), GI symptoms (n=NR), Neurological symptoms (n=2) | | PCR positivity (n=10), Serology positivity (n=1), Abnormal echocardiography (n=2) | Steroids (n=3), IVIG (n=10), anticoagulants (n=7), inotropes (n=4), antivirals (n=1), Mechanical ventilation (n=0)  Recovered (n=10)  ICU admission (n=0), Deaths (n=0), |
| Sadiq 2021 | Pakistan | LMIC | 8 |  | 7 | Contact history (n=NR)  Comorbidities (n=0) | Fever (n=8), Conjunctivitis (n=7), Erythema and cracking of lips, tongue, oral mucosa (n=2), Cervical lymphadenopathy (n=2), Erythema and edema of hands and feet (n=8), Rash (n=5), Complete KD (n=2), Incomplete KD (n=4), Myocarditis (n=2), Shock (n=3), Diarrhea (n=2), Vomiting (n=2), GI symptoms (n=6), Neurological symptoms (n=3) | | PCR positivity (n=3), Serology positivity (n=8), Abnormal echocardiography (n=6) | Steroids (n=3), IVIG (n=7), anticoagulants (n=3), inotropes (n=1), antivirals (n=NR), Mechanical ventilation (n=1)  Recovered (n=7)  ICU admission (n=2), Deaths (n=1), |
| Sagar S. Lad 2021 | India | LMIC | 4 |  | 2 | Contact history (n=NR)  Comorbidities (n=0) | Fever (n=1), Conjunctivitis (n=0), Erythema and cracking of lips, tongue, oral mucosa (n=NR), Cervical lymphadenopathy (n=NR), Erythema and edema of hands and feet (n=NR), Rash (n=NR), Complete KD (n=0), Incomplete KD (n=1), Myocarditis (n=0), Shock (n=2), Diarrhea (n=0), Vomiting (n=0), GI symptoms (n=0), Neurological symptoms (n=3) | | PCR positivity (n=NR), Serology positivity (n=NR), Abnormal echocardiography (n=NR) | Steroids (n=4), IVIG (n=0), anticoagulants (n=NR), inotropes (n=0), antivirals (n=NR), Mechanical ventilation (n=0)  Recovered (n=NR)  ICU admission (n=0), Deaths (n=NR), |
| Borgi 2021 | Tunisia | LMIC | 8 |  | 7 | Contact history (n=4)  Comorbidities (n=0) | Fever (n=8), Conjunctivitis (n=7), Erythema and cracking of lips, tongue, oral mucosa (n=5), Cervical lymphadenopathy (n=1), Erythema and edema of hands and feet (n=0), Rash (n=7), Complete KD (n=0), Incomplete KD (n=0), Myocarditis (n=7), Shock (n=3), Diarrhea (n=7), Vomiting (n=3), GI symptoms (n=8), Neurological symptoms (n=3) | | PCR positivity (n=1), Serology positivity (n=8), Abnormal echocardiography (n=1) | Steroids (n=8), IVIG (n=8), anticoagulants (n=5), inotropes (n=8), antivirals (n=0), Mechanical ventilation (n=3)  Recovered (n=8)  ICU admission (n=8), Deaths (n=0), |
| Yasemin Ozsurekci 2021 | Turkey | LMIC | 30 |  | 16 | Contact history (n=26)  Comorbidities (n=3) | Fever (n=30), Conjunctivitis (n=29), Erythema and cracking of lips, tongue, oral mucosa (n=NR), Cervical lymphadenopathy (n=NR), Erythema and edema of hands and feet (n=23), Rash (n=21), Complete KD (n=NR), Incomplete KD (n=NR), Myocarditis (n=2), Shock (n=NR), Diarrhea (n=11), Vomiting (n=21), GI symptoms (n=NR), Neurological symptoms (n=27) | | PCR positivity (n=3), Serology positivity (n=28), Abnormal echocardiography (n=16) | Steroids (n=29), IVIG (n=30), anticoagulants (n=NR), inotropes (n=NR), antivirals (n=0), Mechanical ventilation (n=3)  Recovered (n=28)  ICU admission (n=14), Deaths (n=0), |
| Shobhna Gupta DCH 2021 | India | LMIC | 20 |  | 12 | Contact history (n=NR)  Comorbidities (n=11) | Fever (n=20), Conjunctivitis (n=4), Erythema and cracking of lips, tongue, oral mucosa (n=NR), Cervical lymphadenopathy (n=NR), Erythema and edema of hands and feet (n=7), Rash (n=7), Complete KD (n=2), Incomplete KD (n=2), Myocarditis (n=3), Shock (n=13), Diarrhea (n=5), Vomiting (n=5), GI symptoms (n=5), Neurological symptoms (n=16) | | PCR positivity (n=40), Serology positivity (n=1), Abnormal echocardiography (n=NR) | Steroids (n=16), IVIG (n=4), anticoagulants (n=NR), inotropes (n=NR), antivirals (n=1), Mechanical ventilation (n=13)  Recovered (n=8)  ICU admission (n=18), Deaths (n=12), |
| Lakshmi Shobhavat 2021 | India | LMIC | 21 |  | 10 | Contact history (n=NR)  Comorbidities (n=1) | Fever (n=21), Conjunctivitis (n=9), Erythema and cracking of lips, tongue, oral mucosa (n=NR), Cervical lymphadenopathy (n=0), Erythema and edema of hands and feet (n=2), Rash (n=7), Complete KD (n=NR), Incomplete KD (n=NR), Myocarditis (n=2), Shock (n=20), Diarrhea (n=0), Vomiting (n=0), GI symptoms (n=16), Neurological symptoms (n=1) | | PCR positivity (n=8), Serology positivity (n=16), Abnormal echocardiography (n=14) | Steroids (n=18), IVIG (n=11), anticoagulants (n=21), inotropes (n=19), antivirals (n=5), Mechanical ventilation (n=13)  Recovered (n=18)  ICU admission (n=21), Deaths (n=3), |
| Pignatelli 2021 | Latin America | LMIC | 98 |  | 49 | Contact history (n=36)  Comorbidities (n=21) | Fever (n=67), Conjunctivitis (n=0), Erythema and cracking of lips, tongue, oral mucosa (n=62), Cervical lymphadenopathy (n=0), Erythema and edema of hands and feet (n=0), Rash (n=0), Complete KD (n=0), Incomplete KD (n=0), Myocarditis (n=18), Shock (n=0), Diarrhea (n=0), Vomiting (n=0), GI symptoms (n=69), Neurological symptoms (n=15) | | PCR positivity (n=51), Serology positivity (n=41), Abnormal echocardiography (n=60) | Steroids (n=0), IVIG (n=68), anticoagulants (n=26), inotropes (n=15), antivirals (n=0), Mechanical ventilation (n=13)  Recovered (n=98)  ICU admission (n=64), Deaths (n=0), |
| Vukomanovic 2021 | Serbia | LMIC | 3 |  | 3 | Contact history (n=1)  Comorbidities (n=0) | Fever (n=3), Conjunctivitis (n=3), Erythema and cracking of lips, tongue, oral mucosa (n=0), Cervical lymphadenopathy (n=0), Erythema and edema of hands and feet (n=3), Rash (n=3), Complete KD (n=0), Incomplete KD (n=1), Myocarditis (n=3), Shock (n=2), Diarrhea (n=1), Vomiting (n=1), GI symptoms (n=3), Neurological symptoms (n=0) | | PCR positivity (n=0), Serology positivity (n=1), Abnormal echocardiography (n=3) | Steroids (n=2), IVIG (n=3), anticoagulants (n=1), inotropes (n=2), antivirals (n=0), Mechanical ventilation (n=0)  Recovered (n=3)  ICU admission (n=0), Deaths (n=0), |
| Elif Celikel 2021 | Turkey | LMIC | 33 |  | 21 | Contact history (n=0)  Comorbidities (n=0) | Fever (n=33), Conjunctivitis (n=15), Erythema and cracking of lips, tongue, oral mucosa (n=0), Cervical lymphadenopathy (n=6), Erythema and edema of hands and feet (n=0), Rash (n=17), Complete KD (n=0), Incomplete KD (n=21), Myocarditis (n=0), Shock (n=0), Diarrhea (n=20), Vomiting (n=14), GI symptoms (n=0), Neurological symptoms (n=11) | | PCR positivity (n=2), Serology positivity (n=33), Abnormal echocardiography (n=33) | Steroids (n=33), IVIG (n=33), anticoagulants (n=0), inotropes (n=24), antivirals (n=0), Mechanical ventilation (n=7)  Recovered (n=31)  ICU admission (n=33), Deaths (n=2), |
| Riphagen S 2021 | UK | HIC | 8 |  | 5 | Contact history (n=4)  Comorbidities (n=0) | Fever (n=8), Conjunctivitis (n=5), Erythema and cracking of lips, tongue, oral mucosa (n=NR), Cervical lymphadenopathy (n=NR), Erythema and edema of hands and feet (n=NR), Rash (n=4), Complete KD (n=NR), Incomplete KD (n=NR), Myocarditis (n=0), Shock (n=8), Diarrhea (n=7), Vomiting (n=4), GI symptoms (n=NR), Neurological symptoms (n=2) | | PCR positivity (n=8), Serology positivity (n=8), Abnormal echocardiography (n=5) | Steroids (n=5), IVIG (n=8), anticoagulants (n=7), inotropes (n=2), antivirals (n=0), Mechanical ventilation (n=7)  Recovered (n=7)  ICU admission (n=8), Deaths (n=1), |
| Lucio Verdoni 2021 | Italy | HIC | 10 |  | 7 | Contact history (n=5)  Comorbidities (n=0) | Fever (n=10), Conjunctivitis (n=8), Erythema and cracking of lips, tongue, oral mucosa (n=5), Cervical lymphadenopathy (n=1), Erythema and edema of hands and feet (n=5), Rash (n=8), Complete KD (n=5), Incomplete KD (n=5), Myocarditis (n=0), Shock (n=5), Diarrhea (n=NR), Vomiting (n=NR), GI symptoms (n=NR), Neurological symptoms (n=4) | | PCR positivity (n=10), Serology positivity (n=10), Abnormal echocardiography (n=6) | Steroids (n=8), IVIG (n=10), anticoagulants (n=8), inotropes (n=2), antivirals (n=0), Mechanical ventilation (n=NR)  Recovered (n=10)  ICU admission (n=NR), Deaths (n=0), |
| Belhadjer Z 2021 | France | HIC | 35 |  | 18 | Contact history (n=13)  Comorbidities (n=10) | Fever (n=35), Conjunctivitis (n=NR), Erythema and cracking of lips, tongue, oral mucosa (n=NR), Cervical lymphadenopathy (n=21), Erythema and edema of hands and feet (n=NR), Rash (n=20), Complete KD (n=NR), Incomplete KD (n=NR), Myocarditis (n=NR), Shock (n=28), Diarrhea (n=NR), Vomiting (n=NR), GI symptoms (n=29), Neurological symptoms (n=11) | | PCR positivity (n=31), Serology positivity (n=NR), Abnormal echocardiography (n=10) | Steroids (n=12), IVIG (n=25), anticoagulants (n=23), inotropes (n=28), antivirals (n=0), Mechanical ventilation (n=24)  Recovered (n=28)  ICU admission (n=35), Deaths (n=0), |
| Toubiana Julie 2021 | France | HIC | 21 |  | 9 | Contact history (n=10)  Comorbidities (n=0) | Fever (n=21), Conjunctivitis (n=17), Erythema and cracking of lips, tongue, oral mucosa (n=16), Cervical lymphadenopathy (n=12), Erythema and edema of hands and feet (n=10), Rash (n=16), Complete KD (n=11), Incomplete KD (n=10), Myocarditis (n=16), Shock (n=NR), Diarrhea (n=NR), Vomiting (n=NR), GI symptoms (n=21), Neurological symptoms (n=18) | | PCR positivity (n=21), Serology positivity (n=21), Abnormal echocardiography (n=8) | Steroids (n=10), IVIG (n=21), anticoagulants (n=21), inotropes (n=15), antivirals (n=0), Mechanical ventilation (n=11)  Recovered (n=21)  ICU admission (n=17), Deaths (n=0), |
| Grimaud M 2021 | France | HIC | 20 |  | 10 | Contact history (n=NR)  Comorbidities (n=0) | Fever (n=20), Conjunctivitis (n=6), Erythema and cracking of lips, tongue, oral mucosa (n=5), Cervical lymphadenopathy (n=2), Erythema and edema of hands and feet (n=0), Rash (n=10), Complete KD (n=NR), Incomplete KD (n=NR), Myocarditis (n=20), Shock (n=20), Diarrhea (n=NR), Vomiting (n=NR), GI symptoms (n=NR), Neurological symptoms (n=NR) | | PCR positivity (n=20), Serology positivity (n=20), Abnormal echocardiography (n=20) | Steroids (n=2), IVIG (n=20), anticoagulants (n=NR), inotropes (n=19), antivirals (n=0), Mechanical ventilation (n=8)  Recovered (n=6)  ICU admission (n=20), Deaths (n=0), |
| Whittaker E 2021 | UK | HIC | 58 |  | 25 | Contact history (n=NR)  Comorbidities (n=7) | Fever (n=58), Conjunctivitis (n=26), Erythema and cracking of lips, tongue, oral mucosa (n=17), Cervical lymphadenopathy (n=9), Erythema and edema of hands and feet (n=9), Rash (n=30), Complete KD (n=13), Incomplete KD (n=NR), Myocarditis (n=29), Shock (n=29), Diarrhea (n=NR), Vomiting (n=NR), GI symptoms (n=NR), Neurological symptoms (n=20) | | PCR positivity (n=58), Serology positivity (n=46), Abnormal echocardiography (n=8) | Steroids (n=37), IVIG (n=41), anticoagulants (n=NR), inotropes (n=27), antivirals (n=NR), Mechanical ventilation (n=25)  Recovered (n=57)  ICU admission (n=29), Deaths (n=1), |
| Blondiaux E 2021 | France | HIC | 4 |  | 1 | Contact history (n=0)  Comorbidities (n=0) | Fever (n=3), Conjunctivitis (n=2), Erythema and cracking of lips, tongue, oral mucosa (n=1), Cervical lymphadenopathy (n=1), Erythema and edema of hands and feet (n=0), Rash (n=4), Complete KD (n=NR), Incomplete KD (n=NR), Myocarditis (n=4), Shock (n=4), Diarrhea (n=2), Vomiting (n=2), GI symptoms (n=NR), Neurological symptoms (n=NR) | | PCR positivity (n=4), Serology positivity (n=4), Abnormal echocardiography (n=3) | Steroids (n=3), IVIG (n=4), anticoagulants (n=3), inotropes (n=3), antivirals (n=NR), Mechanical ventilation (n=1)  Recovered (n=4)  ICU admission (n=4), Deaths (n=0), |
| Ramcharan T 2021 | UK | HIC | 15 |  | 11 | Contact history (n=3)  Comorbidities (n=4) | Fever (n=15), Conjunctivitis (n=NR), Erythema and cracking of lips, tongue, oral mucosa (n=NR), Cervical lymphadenopathy (n=NR), Erythema and edema of hands and feet (n=NR), Rash (n=NR), Complete KD (n=7), Incomplete KD (n=8), Myocarditis (n=15), Shock (n=NR), Diarrhea (n=NR), Vomiting (n=15), GI symptoms (n=13), Neurological symptoms (n=NR) | | PCR positivity (n=4), Serology positivity (n=12), Abnormal echocardiography (n=14) | Steroids (n=5), IVIG (n=10), anticoagulants (n=11), inotropes (n=10), antivirals (n=0), Mechanical ventilation (n=4)  Recovered (n=15)  ICU admission (n=10), Deaths (n=0), |
| Ng KF 2021 | UK | HIC | 3 |  | 2 | Contact history (n=0)  Comorbidities (n=0) | Fever (n=3), Conjunctivitis (n=3), Erythema and cracking of lips, tongue, oral mucosa (n=2), Cervical lymphadenopathy (n=3), Erythema and edema of hands and feet (n=3), Rash (n=3), Complete KD (n=3), Incomplete KD (n=0), Myocarditis (n=3), Shock (n=3), Diarrhea (n=NR), Vomiting (n=NR), GI symptoms (n=3), Neurological symptoms (n=NR) | | PCR positivity (n=3), Serology positivity (n=3), Abnormal echocardiography (n=2) | Steroids (n=1), IVIG (n=2), anticoagulants (n=2), inotropes (n=3), antivirals (n=0), Mechanical ventilation (n=1)  Recovered (n=3)  ICU admission (n=3), Deaths (n=0), |
| Hameed S 2021 | UK | HIC | 35 |  | 27 | Contact history (n=NR)  Comorbidities (n=NR) | Fever (n=33), Conjunctivitis (n=9), Erythema and cracking of lips, tongue, oral mucosa (n=NR), Cervical lymphadenopathy (n=NR), Erythema and edema of hands and feet (n=NR), Rash (n=13), Complete KD (n=NR), Incomplete KD (n=NR), Myocarditis (n=15), Shock (n=21), Diarrhea (n=NR), Vomiting (n=NR), GI symptoms (n=30), Neurological symptoms (n=NR) | | PCR positivity (n=35), Serology positivity (n=30), Abnormal echocardiography (n=18) | Steroids (n=30), IVIG (n=30), anticoagulants (n=NR), inotropes (n=20), antivirals (n=NR), Mechanical ventilation (n=7)  Recovered (n=NR)  ICU admission (n=24), Deaths (n=NR), |
| Francesco Licciardi 2021 | Italy | HIC | 2 |  | 2 | Contact history (n=1)  Comorbidities (n=1) | Fever (n=2), Conjunctivitis (n=2), Erythema and cracking of lips, tongue, oral mucosa (n=2), Cervical lymphadenopathy (n=2), Erythema and edema of hands and feet (n=2), Rash (n=2), Complete KD (n=2), Incomplete KD (n=0), Myocarditis (n=1), Shock (n=2), Diarrhea (n=NR), Vomiting (n=NR), GI symptoms (n=2), Neurological symptoms (n=NR) | | PCR positivity (n=2), Serology positivity (n=2), Abnormal echocardiography (n=2) | Steroids (n=2), IVIG (n=1), anticoagulants (n=NR), inotropes (n=0), antivirals (n=2), Mechanical ventilation (n=NR)  Recovered (n=2)  ICU admission (n=0), Deaths (n=0), |
| Ouldali N 2021 | France | HIC | 10 |  | 4 | Contact history (n=7)  Comorbidities (n=NR) | Fever (n=NR), Conjunctivitis (n=NR), Erythema and cracking of lips, tongue, oral mucosa (n=NR), Cervical lymphadenopathy (n=NR), Erythema and edema of hands and feet (n=NR), Rash (n=NR), Complete KD (n=5), Incomplete KD (n=5), Myocarditis (n=6), Shock (n=4), Diarrhea (n=NR), Vomiting (n=NR), GI symptoms (n=NR), Neurological symptoms (n=NR) | | PCR positivity (n=9), Serology positivity (n=6), Abnormal echocardiography (n=6) | Steroids (n=5), IVIG (n=9), anticoagulants (n=NR), inotropes (n=5), antivirals (n=NR), Mechanical ventilation (n=0)  Recovered (n=10)  ICU admission (n=6), Deaths (n=0), |
| Bautista Rodriguez 2021 | 33 participating European, Asian, and American hospitals | HIC | 183 |  | 109 | Contact history (n=NR)  Comorbidities (n=59) | Fever (n=124), Conjunctivitis (n=117), Erythema and cracking of lips, tongue, oral mucosa (n=120), Cervical lymphadenopathy (n=72), Erythema and edema of hands and feet (n=NR), Rash (n=NR), Complete KD (n=27), Incomplete KD (n=78), Myocarditis (n=NR), Shock (n=79), Diarrhea (n=NR), Vomiting (n=NR), GI symptoms (n=117), Neurological symptoms (n=22) | | PCR positivity (n=43), Serology positivity (n=95), Abnormal echocardiography (n=121) | Steroids (n=105), IVIG (n=163), anticoagulants (n=124), inotropes (n=72), antivirals (n=5), Mechanical ventilation (n=43)  Recovered (n=180)  ICU admission (n=90), Deaths (n=3), |
| Carbajal R 2021 | France | HIC | 7 |  | 3 | Contact history (n=5)  Comorbidities (n=NR) | Fever (n=7), Conjunctivitis (n=4), Erythema and cracking of lips, tongue, oral mucosa (n=6), Cervical lymphadenopathy (n=1), Erythema and edema of hands and feet (n=4), Rash (n=6), Complete KD (n=2), Incomplete KD (n=5), Myocarditis (n=6), Shock (n=NR), Diarrhea (n=3), Vomiting (n=5), GI symptoms (n=NR), Neurological symptoms (n=NR) | | PCR positivity (n=7), Serology positivity (n=7), Abnormal echocardiography (n=6) | Steroids (n=4), IVIG (n=7), anticoagulants (n=NR), inotropes (n=5), antivirals (n=NR), Mechanical ventilation (n=3)  Recovered (n=7)  ICU admission (n=7), Deaths (n=0), |
| Caro Paton L 2021 | Spain | HIC | 12 |  | 8 | Contact history (n=NR)  Comorbidities (n=NR) | Fever (n=12), Conjunctivitis (n=5), Erythema and cracking of lips, tongue, oral mucosa (n=5), Cervical lymphadenopathy (n=5), Erythema and edema of hands and feet (n=5), Rash (n=5), Complete KD (n=0), Incomplete KD (n=5), Myocarditis (n=4), Shock (n=8), Diarrhea (n=9), Vomiting (n=9), GI symptoms (n=NR), Neurological symptoms (n=NR) | | PCR positivity (n=11), Serology positivity (n=6), Abnormal echocardiography (n=4) | Steroids (n=12), IVIG (n=3), anticoagulants (n=12), inotropes (n=9), antivirals (n=1), Mechanical ventilation (n=2)  Recovered (n=12)  ICU admission (n=12), Deaths (n=0), |
| Moraleda 2021 | Spain | HIC | 31 |  | 18 | Contact history (n=16)  Comorbidities (n=10) | Fever (n=30), Conjunctivitis (n=0), Erythema and cracking of lips, tongue, oral mucosa (n=0), Cervical lymphadenopathy (n=4), Erythema and edema of hands and feet (n=0), Rash (n=23), Complete KD (n=12), Incomplete KD (n=12), Myocarditis (n=15), Shock (n=15), Diarrhea (n=0), Vomiting (n=0), GI symptoms (n=27), Neurological symptoms (n=10) | | PCR positivity (n=17), Serology positivity (n=19), Abnormal echocardiography (n=31) | Steroids (n=21), IVIG (n=20), anticoagulants (n=NR), inotropes (n=NR), antivirals (n=9), Mechanical ventilation (n=6)  Recovered (n=30)  ICU admission (n=20), Deaths (n=1), |
| Davies 2021 | UK | HIC | 78 |  | 52 | Contact history (n=8)  Comorbidities (n=17) | Fever (n=78), Conjunctivitis (n=23), Erythema and cracking of lips, tongue, oral mucosa (n=0), Cervical lymphadenopathy (n=0), Erythema and edema of hands and feet (n=0), Rash (n=35), Complete KD (n=0), Incomplete KD (n=0), Myocarditis (n=0), Shock (n=68), Diarrhea (n=50), Vomiting (n=49), GI symptoms (n=70), Neurological symptoms (n=0) | | PCR positivity (n=17), Serology positivity (n=9), Abnormal echocardiography (n=28) | Steroids (n=57), IVIG (n=59), anticoagulants (n=7), inotropes (n=65), antivirals (n=1), Mechanical ventilation (n=36)  Recovered (n=76)  ICU admission (n=78), Deaths (n=2), |
| Okarska Napierala 2021 | Poland | HIC | 39 |  | 29 | Contact history (n=6)  Comorbidities (n=6) | Fever (n=39), Conjunctivitis (n=26), Erythema and cracking of lips, tongue, oral mucosa (n=30), Cervical lymphadenopathy (n=20), Erythema and edema of hands and feet (n=23), Rash (n=34), Complete KD (n=20), Incomplete KD (n=14), Myocarditis (n=6), Shock (n=1), Diarrhea (n=13), Vomiting (n=15), GI symptoms (n=24), Neurological symptoms (n=30) | | PCR positivity (n=1), Serology positivity (n=6), Abnormal echocardiography (n=7) | Steroids (n=15), IVIG (n=35), anticoagulants (n=33), inotropes (n=NR), antivirals (n=NR), Mechanical ventilation (n=1)  Recovered (n=30)  ICU admission (n=1), Deaths (n=0), |
| Torres 2021 | Chile | HIC | 27 |  | 14 | Contact history (n=9)  Comorbidities (n=7) | Fever (n=27), Conjunctivitis (n=13), Erythema and cracking of lips, tongue, oral mucosa (n=11), Cervical lymphadenopathy (n=0), Erythema and edema of hands and feet (n=7), Rash (n=14), Complete KD (n=4), Incomplete KD (n=18), Myocarditis (n=4), Shock (n=0), Diarrhea (n=17), Vomiting (n=13), GI symptoms (n=0), Neurological symptoms (n=0) | | PCR positivity (n=14), Serology positivity (n=10), Abnormal echocardiography (n=12) | Steroids (n=17), IVIG (n=19), anticoagulants (n=18), inotropes (n=12), antivirals (n=2), Mechanical ventilation (n=12)  Recovered (n=24)  ICU admission (n=16), Deaths (n=0), |
| Garcia Salido 2021 | Spain | HIC | 45 |  | 39 | Contact history (n=0)  Comorbidities (n=8) | Fever (n=43), Conjunctivitis (n=18), Erythema and cracking of lips, tongue, oral mucosa (n=10), Cervical lymphadenopathy (n=2), Erythema and edema of hands and feet (n=31), Rash (n=0), Complete KD (n=0), Incomplete KD (n=0), Myocarditis (n=0), Shock (n=38), Diarrhea (n=30), Vomiting (n=32), GI symptoms (n=3), Neurological symptoms (n=25) | | PCR positivity (n=18), Serology positivity (n=17), Abnormal echocardiography (n=22) | Steroids (n=36), IVIG (n=23), anticoagulants (n=NR), inotropes (n=30), antivirals (n=25), Mechanical ventilation (n=6)  Recovered (n=45)  ICU admission (n=45), Deaths (n=0), |
| Almossa 2021 | Saudi Arabia | HIC | 10 |  | 5 | Contact history (n=6)  Comorbidities (n=5) | Fever (n=10), Conjunctivitis (n=9), Erythema and cracking of lips, tongue, oral mucosa (n=6), Cervical lymphadenopathy (n=5), Erythema and edema of hands and feet (n=5), Rash (n=10), Complete KD (n=2), Incomplete KD (n=2), Myocarditis (n=0), Shock (n=10), Diarrhea (n=7), Vomiting (n=9), GI symptoms (n=0), Neurological symptoms (n=0) | | PCR positivity (n=6), Serology positivity (n=5), Abnormal echocardiography (n=3) | Steroids (n=10), IVIG (n=10), anticoagulants (n=10), inotropes (n=8), antivirals (n=7), Mechanical ventilation (n=2)  Recovered (n=8)  ICU admission (n=9), Deaths (n=2), |
| Ryan C. T. Cheong 2021 | UK | HIC | 50 |  | 33 | Contact history (n=NR)  Comorbidities (n=NR) | Fever (n=50), Conjunctivitis (n=21), Erythema and cracking of lips, tongue, oral mucosa (n=13), Cervical lymphadenopathy (n=8), Erythema and edema of hands and feet (n=NR), Rash (n=1), Complete KD (n=NR), Incomplete KD (n=NR), Myocarditis (n=NR), Shock (n=NR), Diarrhea (n=NR), Vomiting (n=NR), GI symptoms (n=36), Neurological symptoms (n=22) | | PCR positivity (n=12), Serology positivity (n=42), Abnormal echocardiography (n=NR) | Steroids (n=4), IVIG (n=0), anticoagulants (n=NR), inotropes (n=0), antivirals (n=NR), Mechanical ventilation (n=18)  Recovered (n=42)  ICU admission (n=38), Deaths (n=0), |
| Cattaneo 2021 | France | HIC | 11 |  | 8 | Contact history (n=0)  Comorbidities (n=1) | Fever (n=10), Conjunctivitis (n=6), Erythema and cracking of lips, tongue, oral mucosa (n=3), Cervical lymphadenopathy (n=3), Erythema and edema of hands and feet (n=2), Rash (n=1), Complete KD (n=0), Incomplete KD (n=5), Myocarditis (n=6), Shock (n=0), Diarrhea (n=0), Vomiting (n=0), GI symptoms (n=11), Neurological symptoms (n=6) | | PCR positivity (n=6), Serology positivity (n=9), Abnormal echocardiography (n=6) | Steroids (n=3), IVIG (n=4), anticoagulants (n=2), inotropes (n=5), antivirals (n=0), Mechanical ventilation (n=0)  Recovered (n=11)  ICU admission (n=6), Deaths (n=0), |
| Cattalini 2021 | Italy | HIC | 53 |  | NR | Contact history (n=1)  Comorbidities (n=NR) | Fever (n=53), Conjunctivitis (n=27), Erythema and cracking of lips, tongue, oral mucosa (n=14), Cervical lymphadenopathy (n=24), Erythema and edema of hands and feet (n=15), Rash (n=21), Complete KD (n=0), Incomplete KD (n=0), Myocarditis (n=21), Shock (n=0), Diarrhea (n=28), Vomiting (n=14), GI symptoms (n=2), Neurological symptoms (n=8) | | PCR positivity (n=14), Serology positivity (n=31), Abnormal echocardiography (n=15) | Steroids (n=NR), IVIG (n=NR), anticoagulants (n=NR), inotropes (n=NR), antivirals (n=NR), Mechanical ventilation (n=NR)  Recovered (n=NR)  ICU admission (n=NR), Deaths (n=NR), |
| Elisa Fernandez Cooke 2021 | Spain | HIC | 46 |  | 28 | Contact history (n=NR)  Comorbidities (n=NR) | Fever (n=46), Conjunctivitis (n=38), Erythema and cracking of lips, tongue, oral mucosa (n=35), Cervical lymphadenopathy (n=18), Erythema and edema of hands and feet (n=15), Rash (n=NR), Complete KD (n=10), Incomplete KD (n=21), Myocarditis (n=7), Shock (n=17), Diarrhea (n=10), Vomiting (n=5), GI symptoms (n=28), Neurological symptoms (n=4) | | PCR positivity (n=26), Serology positivity (n=21), Abnormal echocardiography (n=22) | Steroids (n=26), IVIG (n=41), anticoagulants (n=0), inotropes (n=4), antivirals (n=0), Mechanical ventilation (n=0)  Recovered (n=46)  ICU admission (n=19), Deaths (n=0), |
| Athina Fouriki 2021 | Switzerland | HIC | 6 |  | 5 | Contact history (n=NR)  Comorbidities (n=0) | Fever (n=6), Conjunctivitis (n=4), Erythema and cracking of lips, tongue, oral mucosa (n=NR), Cervical lymphadenopathy (n=1), Erythema and edema of hands and feet (n=2), Rash (n=4), Complete KD (n=4), Incomplete KD (n=2), Myocarditis (n=1), Shock (n=5), Diarrhea (n=5), Vomiting (n=NR), GI symptoms (n=NR), Neurological symptoms (n=2) | | PCR positivity (n=5), Serology positivity (n=6), Abnormal echocardiography (n=2) | Steroids (n=3), IVIG (n=5), anticoagulants (n=NR), inotropes (n=NR), antivirals (n=5), Mechanical ventilation (n=4)  Recovered (n=NR)  ICU admission (n=6), Deaths (n=NR), |
| Serge Grazioli 2021 | Switzerland | HIC | 4 |  | 4 | Contact history (n=4)  Comorbidities (n=0) | Fever (n=4), Conjunctivitis (n=1), Erythema and cracking of lips, tongue, oral mucosa (n=NR), Cervical lymphadenopathy (n=4), Erythema and edema of hands and feet (n=NR), Rash (n=1), Complete KD (n=NR), Incomplete KD (n=NR), Myocarditis (n=NR), Shock (n=2), Diarrhea (n=NR), Vomiting (n=2), GI symptoms (n=NR), Neurological symptoms (n=1) | | PCR positivity (n=0), Serology positivity (n=4), Abnormal echocardiography (n=1) | Steroids (n=1), IVIG (n=1), anticoagulants (n=4), inotropes (n=NR), antivirals (n=4), Mechanical ventilation (n=2)  Recovered (n=4)  ICU admission (n=2), Deaths (n=0), |
| Susanna Felsentein 2021 | UK | HIC | 29 |  | 20 | Contact history (n=NR)  Comorbidities (n=11) | Fever (n=29), Conjunctivitis (n=20), Erythema and cracking of lips, tongue, oral mucosa (n=12), Cervical lymphadenopathy (n=12), Erythema and edema of hands and feet (n=15), Rash (n=21), Complete KD (n=11), Incomplete KD (n=8), Myocarditis (n=25), Shock (n=8), Diarrhea (n=15), Vomiting (n=21), GI symptoms (n=21), Neurological symptoms (n=5) | | PCR positivity (n=3), Serology positivity (n=14), Abnormal echocardiography (n=19) | Steroids (n=23), IVIG (n=28), anticoagulants (n=)0, inotropes (n=7), antivirals (n=2), Mechanical ventilation (n=NR)  Recovered (n=17)  ICU admission (n=NR), Deaths (n=NR), |
| Israel valverde 2021 | Europe | HIC | 286 |  | 194 | Contact history (n=NR)  Comorbidities (n=16) | Fever (n=276), Conjunctivitis (n=156), Erythema and cracking of lips, tongue, oral mucosa (n=116), Cervical lymphadenopathy (n=76), Erythema and edema of hands and feet (n=73), Rash (n=179), Complete KD (n=NR), Incomplete KD (n=NR), Myocarditis (n=266), Shock (n=115), Diarrhea (n=51), Vomiting (n=51), GI symptoms (n=51), Neurological symptoms (n=43) | | PCR positivity (n=90), Serology positivity (n=157), Abnormal echocardiography (n=103) | Steroids (n=80), IVIG (n=224), anticoagulants (n=108), inotropes (n=80), antivirals (n=20), Mechanical ventilation (n=44)  Recovered (n=278)  ICU admission (n=286), Deaths (n=1), |
| **Aydin F 2021** | **Turkey** | **LMIC** | **26** |  | **16** | **Contact history (n= )**  **Comorbidities (n=)** | **Fever (n=), Conjunctivitis (n=), Erythema and cracking of lips, tongue, oral mucosa (n=), Cervical lymphadenopathy (n=), Erythema and edema of hands and feet (n=), Rash (n=), Complete KD (n=), Incomplete KD (n=), Myocarditis, Shock (n=), Diarrhea (n=), Vomiting (n=), GI symptoms (n=), Neurological symptoms (n=)** | | **PCR positivity (n=), Serology positivity (n=), Abnormal echocardiography (n=)** | **Steroids (n=), IVIG (n=), anticoagulants (n=), inotropes (n=), antivirals (n=), Mechanical ventilation (n=)**  **Recovered (n=)**  **ICU admission (n=0), Deaths (n=),** |
| Choe YJ 2021 | South Korea | HIC | 3 |  | 2 | Contact history (n=3)  Comorbidities (n=0) | Fever (n=3), Conjunctivitis (n=3), Erythema and cracking of lips, tongue, oral mucosa (n=2), Cervical lymphadenopathy (n=0), Erythema and edema of hands and feet (n=2), Rash (n=2), Complete KD (n=0), Incomplete KD (n=3), Myocarditis (n=0), Shock (n=3), Diarrhea (n=3), Vomiting (n=1), GI symptoms (n=3), Neurological symptoms (n=0) | | PCR positivity (n=3), Serology positivity (n=3), Abnormal echocardiography (n=3) | Steroids (n=1), IVIG (n=2), anticoagulants (n=3), inotropes (n=2), antivirals (n=0), Mechanical ventilation (n=0)  Recovered (n=3)  ICU admission (n=2), Deaths (n=0), |
| Mario Sa 2021 | United Kingdom | HIC | 75 |  | 50 | Contact history (n=0)  Comorbidities (n=0) | Fever (n=75), Conjunctivitis (n=2), Erythema and cracking of lips, tongue, oral mucosa (n=2), Cervical lymphadenopathy (n=0), Erythema and edema of hands and feet (n=1), Rash (n=2), Complete KD (n=0), Incomplete KD (n=0), Myocarditis (n=0), Shock (n=2), Diarrhea (n=3), Vomiting (n=4), GI symptoms (n=61), Neurological symptoms (n=9) | | PCR positivity (n=75), Serology positivity (n=51), Abnormal echocardiography (n=53) | Steroids (n=70), IVIG (n=70), anticoagulants (n=7), inotropes (n=0), antivirals (n=0), Mechanical ventilation (n=1)  Recovered (n=74)  ICU admission (n=48), Deaths (n=1), |
| Katrin Mehler 2021 | Germany | HIC | 9 |  | 6 | Contact history (n=0)  Comorbidities (n=3) | Fever (n=8), Conjunctivitis (n=4), Erythema and cracking of lips, tongue, oral mucosa (n=0), Cervical lymphadenopathy (n=0), Erythema and edema of hands and feet (n=1), Rash (n=2), Complete KD (n=0), Incomplete KD (n=0), Myocarditis (n=0), Shock (n=4), Diarrhea (n=0), Vomiting (n=0), GI symptoms (n=7), Neurological symptoms (n=2) | | PCR positivity (n=3), Serology positivity (n=8), Abnormal echocardiography (n=9) | Steroids (n=7), IVIG (n=7), anticoagulants (n=7), inotropes (n=0), antivirals (n=0), Mechanical ventilation (n=4)  Recovered (n=6)  ICU admission (n=4), Deaths (n=3), |
| Abrams JY 2020 | USA | HIC | 1080 |  | 602 | Contact history (n=438)  Comorbidities (n=286) | Fever (n=1080), Conjunctivitis (n=534), Erythema and cracking of lips, tongue, oral mucosa (n=313), Cervical lymphadenopathy (n=167), Erythema and edema of hands and feet (n=207), Rash (n=584), Complete KD (n=121), Incomplete KD (n=113), Myocarditis (n=192), Shock (n=392), Diarrhea (n=573), Vomiting (n=684), GI symptoms (n=560), Neurological symptoms (n=383) | | PCR positivity (n=NR), Serology positivity (n=NR), Abnormal echocardiography (n=968) | Steroids (n=NR), IVIG (n=NR), anticoagulants (n=NR), inotropes (n=NR), antivirals (n=NR), Mechanical ventilation (n=NR)  Recovered (n=1062)  ICU admission (n=648), Deaths (n=18), |
| Alkans G 2021 | Turkey | LMIC | 36 |  | 19 | Contact history (n=12)  Comorbidities (n=1) | Fever (n=36), Conjunctivitis (n=25), Erythema and cracking of lips, tongue, oral mucosa (n=21), Cervical lymphadenopathy (n=5), Erythema and edema of hands and feet (n=7), Rash (n=21), Complete KD (n=25), Incomplete KD (n=3), Myocarditis (n=17), Shock (n=11), Diarrhea (n=10), Vomiting (n=12), GI symptoms (n=NR), Neurological symptoms (n=14) | | PCR positivity (n=1), Serology positivity (n=32), Abnormal echocardiography (n=17) | Steroids (n=36), IVIG (n=36), anticoagulants (n=36), inotropes (n=11), antivirals (n=0), Mechanical ventilation (n=0)  Recovered (n=36)  ICU admission (n=4), Deaths (n=0), |
| Niño-Taravilla 2021 | Chile | HIC | 26 |  | 15 | Contact history (n=NR)  Comorbidities (n=1) | Fever (n=26), Conjunctivitis (n=15), Erythema and cracking of lips, tongue, oral mucosa (n=NR), Cervical lymphadenopathy (n=NR), Erythema and edema of hands and feet (n=NR), Rash (n=NR), Complete KD (n=16), Incomplete KD (n=NR), Myocarditis (n=NR), Shock (n=24), Diarrhea (n=16), Vomiting (n=12), GI symptoms (n=NR), Neurological symptoms (n=NR) | | PCR positivity (n=7), Serology positivity (n=15), Abnormal echocardiography (n=18) | Steroids (n=23), IVIG (n=20), anticoagulants (n=0), inotropes (n=13), antivirals (n=0), Mechanical ventilation (n=10)  Recovered (n=26)  ICU admission (n=26), Deaths (n=0), |
| Tolunay O 2021 | Turkey | LMIC | 52 |  | 20 | Contact history (n=12)  Comorbidities (n=9) | Fever (n=48), Conjunctivitis (n=23), Erythema and cracking of lips, tongue, oral mucosa (n=9), Cervical lymphadenopathy (n=1), Erythema and edema of hands and feet (n=NR), Rash (n=25), Complete KD (n=NR), Incomplete KD (n=11), Myocarditis (n=NR), Shock (n=NR), Diarrhea (n=20), Vomiting (n=25), GI symptoms (n=NR), Neurological symptoms (n=5) | | PCR positivity (n=6), Serology positivity (n=44), Abnormal echocardiography (n=25) | Steroids (n=37), IVIG (n=50), anticoagulants (n=NR), inotropes (n=9), antivirals (n=NR), Mechanical ventilation (n=0)  Recovered (n=52)  ICU admission (n=15), Deaths (n=0), |
| Yamuna Sanil 2021 | USA | HIC | 54 |  | 25 | Contact history (n=NR)  Comorbidities (n=36) | Fever (n=53), Conjunctivitis (n=8), Erythema and cracking of lips, tongue, oral mucosa (n=8), Cervical lymphadenopathy (n=13), Erythema and edema of hands and feet (n=8), Rash (n=26), Complete KD (n=8), Incomplete KD (n=NR), Myocarditis (n=23), Shock (n=28), Diarrhea (n=NR), Vomiting (n=NR), GI symptoms (n=40), Neurological symptoms (n=0) | | PCR positivity (n=24), Serology positivity (n=36), Abnormal echocardiography (n=23) | Steroids (n=12), IVIG (n=43), anticoagulants (n=42), inotropes (n=23), antivirals (n=7), Mechanical ventilation (n=11)  Recovered (n=54)  ICU admission (n=35), Deaths (n=0), |
| Ocal Demir 2021 | Turkey | LMIC | 20 |  | 11 | Contact history (n=18)  Comorbidities (n=2) | Fever (n=20), Conjunctivitis (n=10), Erythema and cracking of lips, tongue, oral mucosa (n=6), Cervical lymphadenopathy (n=2), Erythema and edema of hands and feet (n=5), Rash (n=10), Complete KD (n=NR), Incomplete KD (n=NR), Myocarditis (n=NR), Shock (n=NR), Diarrhea (n=6), Vomiting (n=5), GI symptoms (n=NR), Neurological symptoms (n=0) | | PCR positivity (n=2), Serology positivity (n=15), Abnormal echocardiography (n=17) | Steroids (n=17), IVIG (n=20), anticoagulants (n=18), inotropes (n=9), antivirals (n=0), Mechanical ventilation (n=0)  Recovered (n=20)  ICU admission (n=7), Deaths (n=0), |
| Caro-Domínguez 2021 | Spain | HIC | 37 |  | 21 | Contact history (n=14)  Comorbidities (n=1) | Fever (n=37), Conjunctivitis (n=14), Erythema and cracking of lips, tongue, oral mucosa (n=0), Cervical lymphadenopathy (n=4), Erythema and edema of hands and feet (n=0), Rash (n=20), Complete KD (n=0), Incomplete KD (n=0), Myocarditis (n=0), Shock (n=1), Diarrhea (n=4), Vomiting (n=5), GI symptoms (n=0), Neurological symptoms (n=4) | | PCR positivity (n=15), Serology positivity (n=13), Abnormal echocardiography (n=35) | Steroids (n=0), IVIG (n=0), anticoagulants (n=0), inotropes (n=0), antivirals (n=0), Mechanical ventilation (n=0)  Recovered (n=37)  ICU admission (n=30), Deaths (n=0), |
| Yurttutan S 2021 | Turkey | LMIC | 10 |  | NR | Contact history (n=0)  Comorbidities (n=5) | Fever (n=8), Conjunctivitis (n=0), Erythema and cracking of lips, tongue, oral mucosa (n=0), Cervical lymphadenopathy (n=0), Erythema and edema of hands and feet (n=0), Rash (n=8), Complete KD (n=0), Incomplete KD (n=0), Myocarditis (n=0), Shock (n=1), Diarrhea (n=1), Vomiting (n=3), GI symptoms (n=3), Neurological symptoms (n=1) | | PCR positivity (n=6), Serology positivity (n=6), Abnormal echocardiography (n=2) | Steroids (n=0), IVIG (n=0), anticoagulants (n=0), inotropes (n=0), antivirals (n=0), Mechanical ventilation (n=0)  Recovered (n=8)  ICU admission (n=10), Deaths (n=2), |
| Sugunan S 2021 | India | LMIC | 32 |  | 21 | Contact history (n=0)  Comorbidities (n=4) | Fever (n=32), Conjunctivitis (n=32), Erythema and cracking of lips, tongue, oral mucosa (n=0), Cervical lymphadenopathy (n=0), Erythema and edema of hands and feet (n=0), Rash (n=21), Complete KD (n=0), Incomplete KD (n=0), Myocarditis (n=13), Shock (n=22), Diarrhea (n=0), Vomiting (n=0), GI symptoms (n=27), Neurological symptoms (n=13) | | PCR positivity (n=10), Serology positivity (n=22), Abnormal echocardiography (n=11) | Steroids (n=26), IVIG (n=6), anticoagulants (n=22), inotropes (n=20), antivirals (n=0), Mechanical ventilation (n=4)  Recovered (n=32)  ICU admission (n=30), Deaths (n=0), |
| Rubayet Hasan 2021 | Qatar | HIC | 7 |  | 5 | Contact history (n=NR)  Comorbidities (n=0) | Fever (n=7), Conjunctivitis (n=4), Erythema and cracking of lips, tongue, oral mucosa (n=1), Cervical lymphadenopathy (n=NR), Erythema and edema of hands and feet (n=NR), Rash (n=5), Complete KD (n=NR), Incomplete KD (n=NR), Myocarditis (n=NR), Shock (n=3), Diarrhea (n=3), Vomiting (n=7), GI symptoms (n=NR), Neurological symptoms (n=0) | | PCR positivity (n=3), Serology positivity (n=6), Abnormal echocardiography (n=3) | Steroids (n=6), IVIG (n=7), anticoagulants (n=6), inotropes (n=4), antivirals (n=0), Mechanical ventilation (n=1)  Recovered (n=7)  ICU admission (n=5), Deaths (n=0), |
| A Tiwari 2021 | India | LMIC | 25 |  | 14 | Contact history (n=NR)  Comorbidities (n=4) | Fever (n=25), Conjunctivitis (n=NR), Erythema and cracking of lips, tongue, oral mucosa (n=NR), Cervical lymphadenopathy (n=NR), Erythema and edema of hands and feet (n=NR), Rash (n=NR), Complete KD (n=NR), Incomplete KD (n=18), Myocarditis (n=0), Shock (n=13), Diarrhea (n=17), Vomiting (n=17), GI symptoms (n=17), Neurological symptoms (n=5) | | PCR positivity (n=2), Serology positivity (n=20), Abnormal echocardiography (n=15) | Steroids (n=23), IVIG (n=22), anticoagulants (n=3), inotropes (n=14), antivirals (n=0), Mechanical ventilation (n=5)  Recovered (n=23)  ICU admission (n=20), Deaths (n=2), |
| Haslak 2021 | Turkey | LMIC | 76 |  | 52 | Contact history (n=8)  Comorbidities (n=12) | Fever (n=7), Conjunctivitis (n=0), Erythema and cracking of lips, tongue, oral mucosa (n=0), Cervical lymphadenopathy (n=0), Erythema and edema of hands and feet (n=0), Rash (n=20), Complete KD (n=8), Incomplete KD (n=0), Myocarditis (n=7), Shock (n=0), Diarrhea (n=4), Vomiting (n=5), GI symptoms (n=65), Neurological symptoms (n=14) | | PCR positivity (n=5), Serology positivity (n=59), Abnormal echocardiography (n=46) | Steroids (n=45), IVIG (n=76), anticoagulants (n=71), inotropes (n=22), antivirals (n=7), Mechanical ventilation (n=17)  Recovered (n=75)  ICU admission (n=27), Deaths (n=1), |
| McArdle AJ 2021 | Multicentric | Multicentric | 614 |  | 376 | Contact history (n=NR)  Comorbidities (n=111) | Fever (n=545), Conjunctivitis (n=NR), Erythema and cracking of lips, tongue, oral mucosa (n=NR), Cervical lymphadenopathy (n=NR), Erythema and edema of hands and feet (n=NR), Rash (n=NR), Complete KD (n=NR), Incomplete KD (n=NR), Myocarditis (n=NR), Shock (n=NR), Diarrhea (n=281), Vomiting (n=324), GI symptoms (n=NR), Neurological symptoms (n=164) | | PCR positivity (n=133), Serology positivity (n=424), Abnormal echocardiography (n=NR) | Steroids (n=307), IVIG (n=454), anticoagulants (n=NR), inotropes (n=NR), antivirals (n=NR), Mechanical ventilation (n=NR)  Recovered (n=602)  ICU admission (n=NR), Deaths (n=12), |
| Mohsin SS 2021 | Pakistan | LMIC | 30 |  | 23 | Contact history (n=NR)  Comorbidities (n=NR) | Fever (n=30), Conjunctivitis (n=12), Erythema and cracking of lips, tongue, oral mucosa (n=12), Cervical lymphadenopathy (n=12), Erythema and edema of hands and feet (n=12), Rash (n=12), Complete KD (n=12), Incomplete KD (n=18), Myocarditis (n=10), Shock (n=10), Diarrhea (n=21), Vomiting (n=21), GI symptoms (n=NR), Neurological symptoms (n=11) | | PCR positivity (n=14), Serology positivity (n=7), Abnormal echocardiography (n=10) | Steroids (n=24), IVIG (n=12), anticoagulants (n=23), inotropes (n=22), antivirals (n=NR), Mechanical ventilation (n=12)  Recovered (n=24)  ICU admission (n=27), Deaths (n=6), |
| Chiotos K 2020 | USA | HIC | 6 | 7 | 1 | No comorbidities | Fever (n=6), conjunctivitis (n=2), erythema and cracking of lips, tongue or oral mucosa (n=3), erythema and edema of hands and feet (n=2), rash (n=2) | | PCR positive (n=3), serology IgG positive (n=5) | Steroids (n=6), IVIG (n=4), anticoagulants (n=2), inotropes (n=5), antivirals (n=0), Mechanical ventilation (n=5)  Recovered (n=NR)  ICU admission (n=NR), Deaths (n=NR), |
| Miller J 2020 | USA | HIC | 44 | 7.3 | 20 | Overweight (n=16) | Fever (n=44), conjunctivitis (n=23), erythema and cracking of lips, tongue or oral mucosa (n=23), rash (n=31) | | PCR positive (n=15), serology positive (n=31) | Steroids (n=42), IVIG (n=36), anticoagulants (n=40), inotropes (n=0), antivirals (n=0), Mechanical ventilation (n=1)  Recovered (n=NR)  ICU admission (n=NR), Deaths (n=NR), |
| Cheung EW 2020 | USA | HIC | 17 | 8 | 8 | Asthma (n=3) | Fever (n=17), conjunctivitis (n=11), cervical lymphadenopathy (n=9), erythema and edema of hands and feet (n=6), rash (n=12) | | PCR positive (n=8), serology positive (n=9) | Steroids (n=15), IVIG (n=13), anticoagulants (n=15), inotropes (n=10), antivirals (n=NR), Mechanical ventilation (n=0)  Recovered (n=NR)  ICU admission (n=NR), Deaths (n=NR), |
| Capone CA 2020 | USA | HIC | 33 | 10 | 20 | No comorbidities (n=26, including overweight=2, obese=12),  Asthma (n=5),  Others (n=2) | Fever (n=33) | | PCR positive (n=3), serology positive (n=30) | Steroids (n=23), IVIG (n=33), anticoagulants (n=29), inotropes (n=0), antivirals (n=0), Mechanical ventilation (n=6)  Recovered (n=NR)  ICU admission (n=NR), Deaths (n=NR), |
| Kaushik S 2020 | USA | HIC | 33 | 10 | 20 | Overweight (n=4), obese (n=2),  Asthma (n=5), allergic rhinitis/eczema (n=3), cardiac comorbidities (n=2), hematologic disorders (n=2), others (n=3) | Fever (n=31), conjunctivitis (n=12), rash (n=14) | | PCR positive (n=11), serology positive (n=27) | Steroids (n=17), IVIG (n=18), anticoagulants (n=33), inotropes (n=17), antivirals (n=0), Mechanical ventilation (n=5)  Recovered (n=NR)  ICU admission (n=NR), Deaths (n=NR), |
| Riollano-Cruz M 2020 | USA | HIC | 15 | 12 | 11 | no comorbidities (n=11),  asthma (n=4),  hypothyroidism and non-alcoholic fatty liver disease (n=1) | Fever (n=15), conjunctivitis (n=4), erythema and edema of hands and feet (n=4), rash (n=7) | | PCR positive (n=9), serology positive (n=15) | Steroids (n=3), IVIG (n=12), anticoagulants (n=15), inotropes (n=8), antivirals (n=2), Mechanical ventilation (n=3)  Recovered (n=NR)  ICU admission (n=NR), Deaths (n=NR), |
| Schupper AJ 2020 | USA | HIC | 2 |  | 2 | Tracheomalacia (n=1) | Fever (n=1) | | Serology positive (n=1) | Steroids (n=NR), IVIG (n=NR), anticoagulants (n=NR), inotropes (n=NR), antivirals (n=NR), Mechanical ventilation (n=0)  Recovered (n=NR)  ICU admission (n=NR), Deaths (n=NR), |
| Dufort EM 2020 | USA | HIC | 99 | n/a | 53 | chronic lung disease (n=14), obesity (n=29) | Fever (n=99), conjunctivitis (n=55), cervical lymphadenopathy (n=6), erythema and edema of hands and feet (n=9), rash (n=59) | | PCR positive (n=94), serology positive (n=77) | Steroids (n=63), IVIG (n=69), anticoagulants (n=NR), inotropes (n=61), antivirals (n=NR), Mechanical ventilation (n=10)  Recovered (n=NR)  ICU admission (n=NR), Deaths (n=NR), |
| Feldstein LR 2020 | USA | HIC | 186 | 8.3 | 115 | no comorbidities (n=135),  respiratory disorders (n=33), cardiac comorbidities (n=5),  immunocompromising or autoimmune disorders (n=10), others (n=20);  clinically diagnosed obesity (n=12, out of 153);  BMI-based obesity (n=45, out of 153) | Fever (n=151), conjunctivitis (n=103), erythema and cracking of lips, tongue or oral mucosa (n=78), cervical lymphadenopathy (n=18), rash (n=110), erythema and edema of hands and feet (n=69) | | PCR positive (n=73), serology positive (n=58) | Steroids (n=91), IVIG (n=144), anticoagulants (n=87), inotropes (n=90), antivirals (n=NR), Mechanical ventilation (n=69)  Recovered (n=NR)  ICU admission (n=NR), Deaths (n=NR), |
| Blumfield E 2021 | USA | HIC |  |  |  |  |  | |  | Steroids (n=10), IVIG (n=5), anticoagulants (n=NR), inotropes (n=0), antivirals (n=NR), Mechanical ventilation (n=1)  Recovered (n=NR)  ICU admission (n=NR), Deaths (n=NR), |
| Cantor A 2020 | USA | HIC |  |  |  |  |  | |  | Steroids (n=NR), IVIG (n=NR), anticoagulants (n=NR), inotropes (n=1), antivirals (n=NR), Mechanical ventilation (n=10)  Recovered (n=NR)  ICU admission (n=NR), Deaths (n=NR), |
| Kest H 2020 | USA | HIC |  |  |  |  |  | |  | Steroids (n=3), IVIG (n=2), anticoagulants (n=0), inotropes (n=3) antivirals (n=0), Mechanical ventilation (n=2)  Recovered (n=NR)  ICU admission (n=NR), Deaths (n=NR), |
| Daube A | USA | HIC |  |  |  |  |  | |  | Steroids (n=2), IVIG (n=3), anticoagulants (n=NR), inotropes (n=0), antivirals (n=NR), Mechanical ventilation (n=1)  Recovered (n=NR)  ICU admission (n=NR), Deaths (n=NR), |
| Halepas S 2020 | USA | HIC |  |  |  |  |  | |  | Steroids (n=NR), IVIG (n=NR), anticoagulants (n=NR), inotropes (n=NR), antivirals (n=NR), Mechanical ventilation (n=0)  Recovered (n=NR)  ICU admission (n=NR), Deaths (n=NR), |
| Jonat B 2021 | USA | HIC |  |  |  |  |  | |  | Steroids (n=5), IVIG (n=9), anticoagulants (n=NR), inotropes (n=NR), antivirals (n=NR), Mechanical ventilation (n=0)  Recovered (n=NR)  ICU admission (n=NR), Deaths (n=NR), |
| Basalely A | USA | HIC |  |  |  |  |  | |  | Steroids (n=35), IVIG (n=49), anticoagulants (n=0), inotropes (n=13), antivirals (n=3), Mechanical ventilation (n=5)  Recovered (n=NR)  ICU admission (n=NR), Deaths (n=NR), |
| Abdel-Haq N 2021 | USA | HIC |  |  |  |  |  | |  | Steroids (n=1), IVIG (n=29), anticoagulants (n=33), inotropes (n=17), antivirals (n=2), Mechanical ventilation (n=6)  Recovered (n=NR)  ICU admission (n=NR), Deaths (n=NR), |
| Dionne A 2020 | US | HIC |  |  |  |  |  | |  | Steroids (n=13), IVIG (n=16), anticoagulants (n=14), inotropes (n=7), antivirals (n=NR), Mechanical ventilation (n=1)  Recovered (n=NR)  ICU admission (n=NR), Deaths (n=NR), |
| Diorio C 2020 | US | HIC |  |  |  |  |  | |  | Steroids (n=0), IVIG (n=6), anticoagulants (n=NR), inotropes (n=12), antivirals (n=NR), Mechanical ventilation (n=0)  Recovered (n=NR)  ICU admission (n=NR), Deaths (n=NR), |
| Fenlon Iii EP 2021 | US | HIC |  |  |  |  |  | |  | Steroids (n=NR), IVIG (n=NR), anticoagulants (n=NR), inotropes (n=NR), antivirals (n=NR), Mechanical ventilation (n=0)  Recovered (n=NR)  ICU admission (n=NR), Deaths (n=NR), |
| Fernandes DM 2021 | US | HIC |  |  |  |  |  | |  | Steroids (n=NR), IVIG (n=47), anticoagulants (n=98), inotropes (n=NR), antivirals (n=24), Mechanical ventilation (n=29)  Recovered (n=NR)  ICU admission (n=NR), Deaths (n=NR), |
| Godfred-Cato S 2020 | US | HIC |  |  |  |  |  | |  | Steroids (n=331), IVIG (n=424), anticoagulants (n=233), inotropes (n=NR), antivirals (n=0), Mechanical ventilation (n=69)  Recovered (n=NR)  ICU admission (n=NR), Deaths (n=NR), |
| Heidemann SM 2020 | US | HIC |  |  |  |  |  | |  | Steroids (n=NR), IVIG (n=3), anticoagulants (n=NR), inotropes (n=NR), antivirals (n=3), Mechanical ventilation (n=2)  Recovered (n=NR)  ICU admission (n=NR), Deaths (n=NR), |
| Jhaveri S 2021 | US | HIC |  |  |  |  |  | |  | Steroids (n=3), IVIG (n=12), anticoagulants (n=15), inotropes (n=NR), antivirals (n=NR), Mechanical ventilation (n=1)  Recovered (n=NR)  ICU admission (n=NR), Deaths (n=NR), |
| Lee EH 2020 |  |  |  |  |  |  |  | |  | Steroids (n=NR), IVIG (n=NR), anticoagulants (n=NR), inotropes (n=NR), antivirals (n=NR), Mechanical ventilation (n=NR)  Recovered (n=NR)  ICU admission (n=NR), Deaths (n=NR), |
| Matsubara D 2020 | US | HIC |  |  |  |  |  | |  | Steroids (n=23), IVIG (n=20), anticoagulants (n=19), inotropes (n=NR), antivirals (n=21), Mechanical ventilation (n=8)  Recovered (n=NR)  ICU admission (n=NR), Deaths (n=NR), |
| Minocha PK 2021 | US | HIC |  |  |  |  |  | |  | Steroids (n=18), IVIG (n=33), anticoagulants (n=NR), inotropes (n=NR), antivirals (n=0), Mechanical ventilation (n=0)  Recovered (n=NR)  ICU admission (n=NR), Deaths (n=NR), |
| Sethuraman U 2021 | US | HIC |  |  |  |  |  | |  | Steroids (n=NR), IVIG (n=34), anticoagulants (n=NR), inotropes (n=16), antivirals (n=0), Mechanical ventilation (n=8)  Recovered (n=NR)  ICU admission (n=NR), Deaths (n=NR), |
| Rogo T 2020 | US | HIC |  |  |  |  |  | |  | Steroids (n=0), IVIG (n=2), anticoagulants (n=1), inotropes (n=1), antivirals (n=0), Mechanical ventilation (n=0)  Recovered (n=NR)  ICU admission (n=NR), Deaths (n=NR), |
| Godfred-Cato S 2020 | US | HIC |  |  |  |  |  | |  | Steroids (n=45), IVIG (n=49), anticoagulants (n=21), inotropes (n=16), antivirals (n=0), Mechanical ventilation (n=4)  Recovered (n=NR)  ICU admission (n=NR), Deaths (n=NR), |

NR= Not reported

**Table 4.1. Comparison between MIS-C and KD**

| **Factor** | | | **Study Number** | **Forest plot** | **OR (95%CI)** | **p-value** | **Heterogeneity** $\mathbf{I}^{\boldsymbol{2}}$ **(%)** |  |
| --- | --- | --- | --- | --- | --- | --- | --- | --- |
|  |  |  |  |  |  |  |  |  |
| **Demographic** | | |  |  |  |  |  |  |
|  | **sex** | |  |  |  |  |  |  |
|  |  | woman (ref: male) | 8 |  | 0.97 (0.77-1.22) | 0.787 | 14.760 |  |
|  | **race** | |  |  |  |  |  |  |
|  |  | Black | 4 |  | 2.77 (1.20-6.37) | 0.017 | 0.000 |  |
|  |  | White | 5 |  | 0.47 (0.22-0.99) | 0.047 | 0.000 |  |
|  | **ethnicity** | |  |  |  |  |  |  |
|  |  | Hispanic | 4 |  | 2.54 (0.99-6.53) | 0.053 | 0.000 |  |
| **Clinical characteristics** | | |  |  |  |  |  |  |
|  |  | Extremity changes | 2 |  | 0.35 (0.08-1.55) | 0.164 | 88.635 |  |
|  |  | Conjunctivitis | 2 |  | 0.27 (0.11-0.67) | 0.005 | 82.700 |  |
|  |  | Cervical illness | 2 |  | 0.21 (0.07-0.68) | 0.009 | 89.091 |  |
|  |  | Complete KD | 2 |  | 0.24 (0.17-0.35) | 0.000 | 12.159 |  |
|  |  | Neurologic signs | 3 | 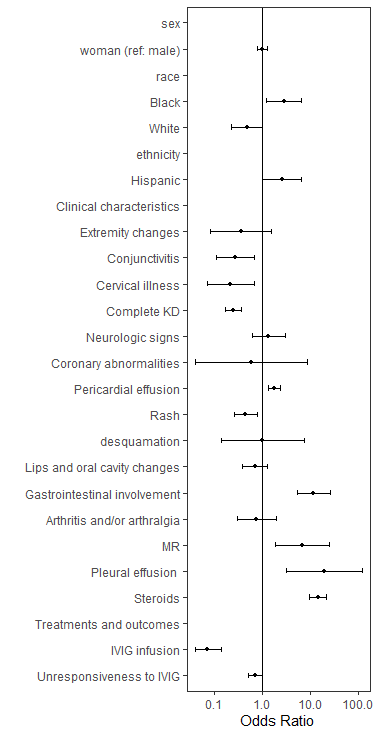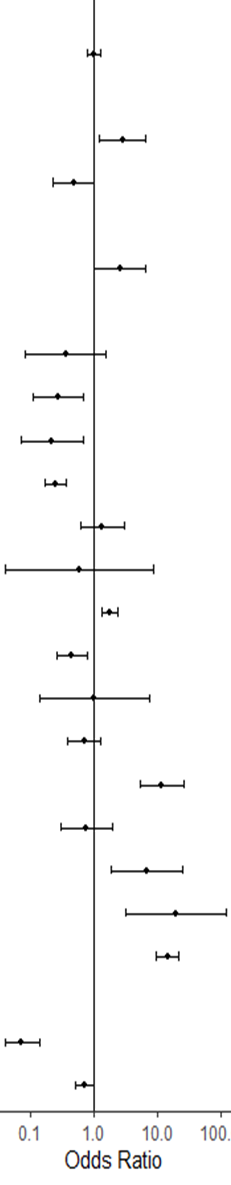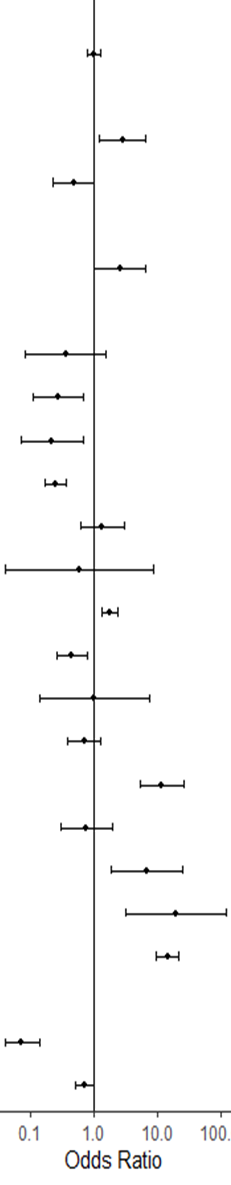 | 1.33 (0.61-2.92) | 0.477 | 78.991 |  |
|  |  | Coronary abnormalities | 2 |  | 0.59 (0.04-8.60) | 0.702 | 93.900 |  |
|  |  | Pericardial effusion | 3 |  | 1.74 (1.28-2.37) | 0.000 | 0.000 |  |
|  |  | Rash | 2 |  | 0.44 (0.26-0.77) | 0.004 | 0.000 |  |
|  |  | desquamation | 2 |  | 1.00 (0.14-7.33) | 0.997 | 63.831 |  |
|  |  | Lips and oral cavity changes | 2 |  | 0.69 (0.38-1.27) | 0.233 | 0.000 |  |
|  |  | Gastrointestinal involvement | 2 |  | 11.44 (5.18-25.26) | 0.000 | 51.044 |  |
|  |  | Arthritis and/or arthralgia | 2 |  | 0.75 (0.29-1.91) | 0.544 | 0.000 |  |
|  |  | MR | 2 |  | 6.62 (1.81-24.20) | 0.004 | 0.000 |  |
|  |  | Pleural effusion | 2 |  | 19.21 (3.10-119.16) | 0.002 | 0.000 |  |
|  |  | Steroids | 2 |  | 14.28 (9.45-21.57) | 0.000 | 82.652 |  |
| **Treatments and outcomes** | | |  |  |  |  |  |  |
|  |  | IVIG infusion | 3 |  | 0.07 (0.04-0.14) | 0.000 | 0.000 |  |
|  |  | Unresponsiveness to IVIG | 2 |  | 0.71 (0.51-1.00) | 0.053 | 0.000 |  |


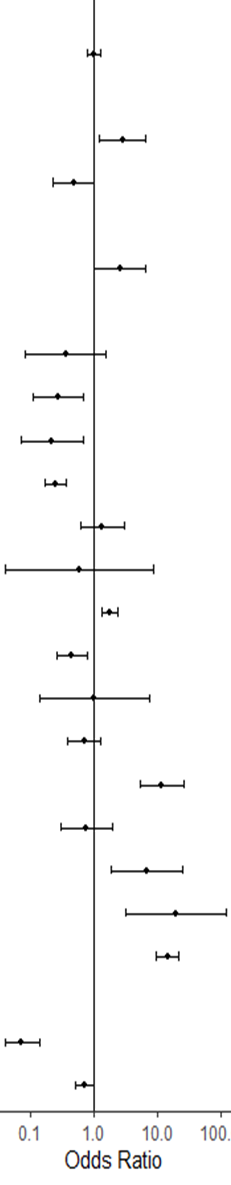

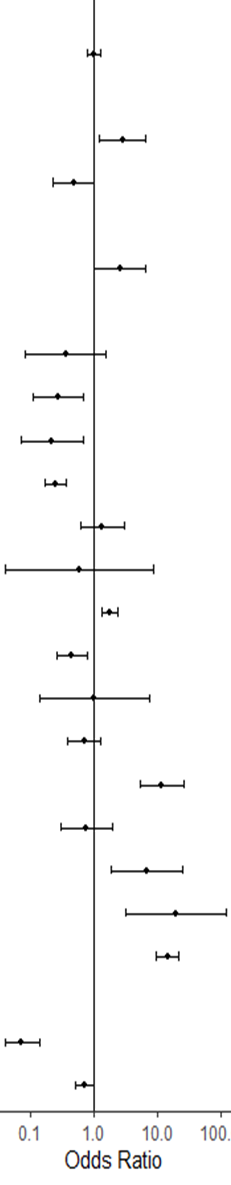


| **Risk Factor** | **Study Number** | **Forest plot** | **OR (95%CI)** | **p-value** | **Heterogeneity I^2^ (%)** |
| --- | --- | --- | --- | --- | --- |
| **Sex** |  |  |  |  |  |
| woman (ref: male) | 18 |  | 1.02 (0.86, 1.21) | 0.705 | 0.0 |
| **Ethnity** |  |  |  |  |  |
| Hispanic | 7 |  | 1.29 (0.99, 1.69) | 0.914 | 0.0 |
| Non-Hispanic white | 10 |  | 1.05 (0.79, 1.40) | 0.254 | 20.6 |
| Asian | 4 |  | 0.85 (0.40, 1.79) | 0.576 | 0.0 |
| Black African | 11 |  | 0.63 (0.49, 0.82) | 0.893 | 0.0 |
| **Comorbidity** |  |  |  |  |  |
| any | 10 |  | 1.89 (1.09, 3.30) | 0.002 | 65.4 |
| neuromuscular | 3 |  | 3.10 (1.39, 6.92) | 0.650 | 0.0 |
| cardiovascular | 4 | 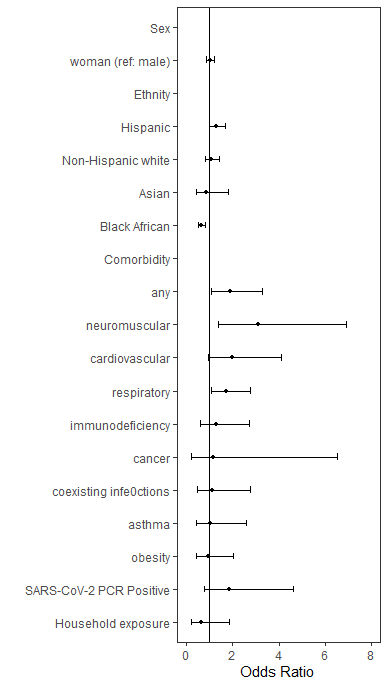 | 1.98 (0.95, 4.12) | 0.577 | 0.0 |
| respiratory | 3 |  | 1.72 (1.08, 2.74) | 0.632 | 0.0 |
| immunodeficiency | 4 |  | 1.27 (0.59, 2.71) | 0.322 | 14.1 |
| cancer | 2 |  | 1.16 (0.20, 6.55) | 0.050 | 73.9 |
| coexisting infe0ctions | 3 |  | 1.13 (0.46, 2.74) | 0.866 | 0.0 |
| asthma | 4 |  | 1.02 (0.40, 2.60) | 0.575 | 0.0 |
| obesity | 5 |  | 0.95 (0.44, 2.03) | 0.007 | 71.7 |
| **SARS-CoV-2 PCR Positive** | 8 |  | 1.86 (0.75, 4.63) | 0.000 | 80.5 |
| **Household exposure** | 3 |  | 0.65 (0.22, 1.87) | 0.023 | 73.5 |

**Table 4.2. The risk factors for developing MIS-C in pediatric COVID-19 population**


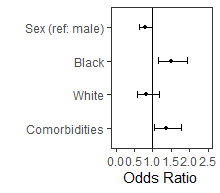
**Table 4.3. The risk factors of ICU admission among MIS-C patients**

| **Factor** | **Study Number** | **Forest plot** | **OR (95%CI)** | **p-value** | **Heterogeneity I2 (%)** |  |
| --- | --- | --- | --- | --- | --- | --- |
|  |  |  |  |  |  |  |
| Sex (ref: male) | 6 | 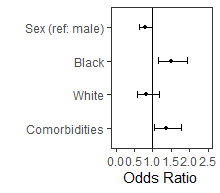 | 0.80 (0.64-0.98) | 0.034 | 34.338 |  |
| Black | 2 |  | 1.50 (1.15-1.95) | 0.003 | 0.000 |  |
| White | 2 |  | 0.83 (0.58-1.17) | 0.283 | 0.000 |  |
| Comorbidities | 4 |  | 1.36 (1.04-1.78) | 0.023 | 0.000 |  |

**Table 5a: Quality assessment of studies included in the descriptive meta-analysis (N=66)**

| Study and year | Was the study question or objective clearly stated? | Was the study population clearly and fully described, including a case definition? | Were the cases consecutive? | Were the subjects comparable? | Was the intervention clearly described? | Were the outcome measures clearly defined, valid, reliable, and implemented consistently across all study participants? | Was the length of follow-up adequate? | Were the statistical methods well described? | Were the results well described? | Overall Quality (Good, fair or poor) |
| --- | --- | --- | --- | --- | --- | --- | --- | --- | --- | --- |
| Aydin 2021 | Yes | Yes | No | Yes | NA | Yes | Yes | Yes | Yes | Good |
| Akca 2021 | Yes | Yes | No | Yes | NA | Yes | Yes | Yes | Yes | Good |
| Antunez- Montez 2021 | Yes | Yes | Yes | Yes | NA | Yes | Yes | Yes | Yes | Good |
| de Farias 2020 | Yes | Yes | Yes | Yes | NA | Yes | Yes | Yes | Yes | Good |
| Mamishi 2020 | Yes | Yes | Yes | Yes | NA | Yes | Yes | Yes | Yes | Good |
| Jain 2020 | Yes | Yes | Yes | Yes | NA | Yes | Yes | Yes | Yes | Good |
| Webb 2020 | Yes | Yes | Yes | Yes | NA | Yes | No | No | No | Fair |
| Shahbaznejad 2020 | Yes | Yes | Yes | Yes | NA | Yes | Yes | Yes | Yes | Good |
| Lima-setta 2020 | Yes | Yes | Yes | Yes | NA | Yes | Yes | Yes | Yes | Good |
| Dhanalakshmi 2020 | Yes | Yes | Yes | Yes | NA | Yes | Yes | Yes | Yes | Good |
| Falah 2020 | Yes | Yes | Yes | Yes | NA | Yes | Yes | Yes | Yes | Good |
| Sadiq 2020 | Yes | Yes | No | Yes | NA | Yes | Yes | Yes | Yes | Good |
| Sagar 2021 | Yes | Yes | No | Yes | NA | Yes | No | No | No | Fair |
| Borgi 2021 | Yes | Yes | No | Yes | NA | Yes | Yes | Yes | Yes | Good |
| Yasemin 2021 | Yes | Yes | Yes | Yes | NA | Yes | No | Yes | No | Good |
| Shobhna 2021 | Yes | Yes | Yes | Yes | NA | Yes | Yes | Yes | Yes | Good |
| Lakshmi 2020 | Yes | Yes | Yes | Yes | NA | Yes | Yes | Yes | Yes | Good |
| Pignatelli 2021 | Yes | Yes | No | Yes | NA | Yes | Yes | Yes | Yes | Good |
| Vukomanovic 2021 | Yes | Yes | No | Yes | NA | Yes | Yes | Yes | Yes | Good |
| Celikel 2021 | Yes | Yes | Yes | Yes | NA | Yes | Yes | Yes | Yes | Good |
| Riphagen 2020 | Yes | No | Yes | Yes | NA | Yes | Yes | Yes | Yes | Good |
| Lucio 2020 | Yes | No | Yes | Yes | NA | Yes | Yes | Yes | Yes | Good |
| Belhadjer 2020 | Yes | Yes | Yes | Yes | NA | Yes | No | Yes | No | Good |
| Toubiana 2020 | Yes | Yes | No | Yes | NA | Yes | Yes | Yes | Yes | Good |
| Grimaud 2020 | Yes | Yes | No | Yes | NA | Yes | No | Yes | No | Fair |
| Whittaker 2020 | Yes | Yes | Yes | Yes | NA | Yes | Yes | Yes | Yes | Good |
| Blondiaux 2020 | Yes | Yes | No | Yes | NA | Yes | Yes | Yes | Yes | Good |
| Ramcharan 2020 | Yes | Yes | Yes | Yes | NA | Yes | Yes | Yes | Yes | Good |
| Ng 2020 | Yes | Yes | No | Yes | NA | Yes | Yes | Yes | Yes | Good |
| Hameed 2020 | Yes | Yes | Yes | Yes | NA | Yes | No | Yes | No | Good |
| Licciardi 2020 | Yes | Yes | No | Yes | NA | Yes | Yes | Yes | Yes | Good |
| Ouldali 2020 | Yes | Yes | No | Yes | NA | Yes | Yes | Yes | Yes | Good |
| Bautista 2020 | Yes | Yes | Yes | Yes | NA | Yes | Yes | Yes | Yes | Good |
| Carbajal 2020 | Yes | Yes | Yes | Yes | NA | Yes | Yes | Yes | Yes | Good |
| Caro-Paton 2021 | Yes | Yes | Yes | Yes | NA | Yes | Yes | Yes | Yes | Good |
| Moraleda 2020 | Yes | Yes | No | Yes | NA | Yes | Yes | Yes | No | Good |
| Davies 2020 | Yes | Yes | Yes | Yes | NA | Yes | Yes | Yes | Yes | Good |
| Okarska-Napierala 2020 | Yes | Yes | Yes | Yes | NA | Yes | No | Yes | No | Good |
| Torres 2020 | Yes | Yes | Yes | Yes | NA | Yes | Yes | Yes | Yes | Good |
| García-Salido 2020 | Yes | Yes | Yes | Yes | NA | Yes | Yes | Yes | Yes | Good |
| Almossa 2020 | Yes | Yes | Yes | Yes | NA | Yes | Yes | Yes | Yes | Good |
| Ryan 2021 | Yes | Yes | No | Yes | NA | Yes | No | Yes | No | Fair |
| Cattaneo 2021 | Yes | Yes | Yes | Yes | NA | Yes | Yes | Yes | Yes | Good |
| Cattalini 2021 | Yes | Yes | Yes | Yes | NA | Yes | No | Yes | No | Good |
| Elisa 2021 | Yes | Yes | Yes | Yes | NA | Yes | Yes | Yes | Yes | Good |
| Athina 2021 | Yes | Yes | No | Yes | NA | Yes | No | Yes | No | Fair |
| Serge 2020 | Yes | Yes | Yes | Yes | NA | Yes | Yes | Yes | Yes | Good |
| Felsenstein 2020 | Yes | Yes | Yes | Yes | NA | Yes | No | Yes | No | Good |
| Valverde 2021 | Yes | Yes | Yes | Yes | NA | Yes | Yes | Yes | Yes | Good |
| Choe 2021 | Yes | Yes | Yes | Yes | NA | Yes | Yes | Yes | Yes | Good |
| Sa 2021 | Yes | Yes | No | Yes | NA | Yes | Yes | Yes | Yes | Good |
| Mehler et 2021 | Yes | Yes | No | Yes | NA | Yes | Yes | Yes | Yes | Good |
| Abrams 2020 | Yes | Yes | Yes | Yes | NA | Yes | Yes | Yes | Yes | Good |
| Alkans 2021 | Yes | Yes | Yes | Yes | NA | Yes | Yes | Yes | Yes | Good |
| Niño-Taravilla 2020 | Yes | Yes | Yes | Yes | NA | Yes | Yes | Yes | Yes | Good |
| Tolunay 2021 | Yes | Yes | Yes | Yes | NA | Yes | Yes | Yes | Yes | Good |
| Sanil 2021 | Yes | Yes | No | Yes | NA | Yes | Yes | Yes | Yes | Good |
| Demir 2020 | Yes | Yes | Yes | Yes | NA | Yes | Yes | Yes | Yes | Good |
| Caro-Domínguez 2021 | Yes | Yes | No | Yes | NA | Yes | Yes | Yes | Yes | Good |
| Yurttutan 2021 | Yes | Yes | No | Yes | NA | Yes | Yes | Yes | Yes | Good |
| Suguna 2021 | Yes | Yes | No | Yes | NA | Yes | Yes | Yes | Yes | Good |
| Hasan 2021 | Yes | Yes | Yes | Yes | NA | Yes | Yes | Yes | Yes | Good |
| Tiwari 2021 | Yes | Yes | Yes | Yes | NA | Yes | Yes | Yes | Yes | Good |
| Haslak 2021 | Yes | Yes | Yes | Yes | NA | Yes | Yes | Yes | Yes | Good |
| McArdle 2021 | Yes | Yes | Yes | Yes | NA | Yes | Yes | Yes | Yes | Good |
| Mohsin 2021 | Yes | Yes | Yes | Yes | NA | Yes | Yes | Yes | Yes | Good |

**Table 5b: Quality assessment of studies included in the risk factor meta-analysis (N=37)**

| Study and year | Was the study question or objective clearly stated? | Was the study population clearly and fully described, including a case definition? | Were the cases consecutive? | Were the subjects comparable? | Was the intervention clearly described? | Were the outcome measures clearly defined, valid, reliable, and implemented consistently across all study participants? | Was the length of follow-up adequate? | Were the statistical methods well described? | Were the results well described? | Overall Quality (Good, fair or poor) |
| --- | --- | --- | --- | --- | --- | --- | --- | --- | --- | --- |
| Aishwarya 2021 | Yes | Yes | Yes | Yes | NA | Yes | Yes | Yes | Yes | Good |
| Alberto 2020 | Yes | Yes | Yes | Yes | NA | Yes | No | Yes | Yes | Good |
| Arnaldo 2020 | Yes | Yes | Yes | Yes | NA | Yes | No | Yes | Yes | Good |
| Bilade 2021 | Yes | Yes | Yes | Yes | NA | Yes | Yes | Yes | Yes | Good |
| Bradley 2020 | Yes | Yes | Yes | Yes | NA | Yes | No | Yes | Yes | Good |
| Caroline 2020 | Yes | Yes | Yes | Yes | NA | Yes | No | Yes | Yes | Good |
| Christina 2020 | Yes | Yes | Yes | Yes | NA | Yes | Yes | Yes | Yes | Good |
| Daisuke 2020 | Yes | Yes | Yes | Yes | NA | Yes | Yes | Yes | Yes | Good |
| Daniel 2021 | Yes | Yes | Yes | Yes | NA | Yes | No | Yes | Yes | Good |
| Danielle 2020 | Yes | Yes | Yes | Yes | NA | Yes | No | Yes | Yes | Good |
| Debashis 2021 | Yes | Yes | No | Yes | NA | Yes | Yes | Yes | Yes | Good |
| Hilary 2020 | Yes | Yes | Yes | Yes | NA | Yes | No | Yes | Yes | Good |
| Jessica 2021 | Yes | Yes | Yes | Yes | NA | Yes | No | Yes | Yes | Good |
| Jonathan 2021 | Yes | Yes | Yes | Yes | NA | Yes | NA | Yes | Yes | Good |
| Joseph 2021 | Yes | Yes | Yes | Yes | NA | Yes | NA | Yes | Yes | Good |
| Juan 2020 | Yes | Yes | Yes | Yes | NA | Yes | NA | Yes | Yes | Good |
| Julie 2020 | Yes | Yes | No | Yes | NA | Yes | No | Yes | Yes | Good |
| Kamila 2021 | Yes | No | No | Yes | NA | Yes | Yes | Yes | Yes | Good |
| Lael 2020 | Yes | Yes | Yes | Yes | NA | Yes | No | Yes | Yes | Good |
| Mansi 2020 | Yes | Yes | Yes | Yes | NA | Yes | Yes | Yes | Yes | Good |
| Marco 2021 | Yes | Yes | Yes | Yes | NA | Yes | Yes | Yes | Yes | Good |
| Maria 2020 | Yes | Yes | Yes | Yes | NA | Yes | No | Yes | Yes | Good |
| Merckx 2021 | Yes | Yes | Yes | Yes | NA | Yes | No | Yes | Yes | Good |
| Mohsin 2021 | Yes | Yes | Yes | Yes | NA | Yes | Yes | Yes | Yes | Good |
| Olivia 2020 | Yes | Yes | Yes | Yes | NA | Yes | Yes | Yes | Yes | Good |
| Omar 2020 | Yes | Yes | Yes | Yes | NA | Yes | No | Yes | Yes | Good |
| Ozsurekci 2021 | Yes | Yes | Yes | Yes | NA | Yes | Yes | Yes | Yes | Good |
| Panupattanapong 2020 | Yes | Yes | No | Yes | NA | Yes | No | Yes | Yes | Good |
| Pui 2020 | Yes | Yes | No | Yes | NA | Yes | Yes | Yes | Yes | Good |
| Rebecca 2021 | Yes | Yes | Yes | Yes | NA | Yes | No | Yes | Yes | Good |
| Rostad 2021 | Yes | Yes | Yes | Yes | NA | Yes | No | Yes | Yes | Good |
| Sergey 2020 | Yes | Yes | Yes | Yes | NA | Yes | No | Yes | Yes | Good |
| Stuart 2020 | Yes | Yes | Yes | Yes | NA | Yes | Yes | Yes | Yes | Good |
| Suzuki 2021 | Yes | Yes | No | Yes | NA | Yes | No | Yes | Yes | Good |
| Venkataraman 2021 | Yes | Yes | Yes | Yes | NA | Yes | No | Yes | Yes | Good |
| Yasemin 2021 | Yes | Yes | Yes | Yes | NA | Yes | Yes | Yes | Yes | Good |
| Zou 2021 | Yes | Yes | No | Yes | NA | Yes | No | Yes | Yes | Good |

**Figure1**: **Flow diagram of the systematic review process**

Records excluded
(n = 2017)

## Eligibility

## Included

Additional records identified through other sources
(n = 22)

Records identified through database searching
(n = 1650)

## Identification

Records after duplicates removed
(n = 2130)

## Screening

Records screened
(n = 2152)

Full-text articles assessed for eligibility
(n =135)

Full-text articles excluded, with reasons
(n = 12)

Studies included in the analysis (n = 123); 66 in descriptive and 37 in risk factor analysis

**Figure 2: Global map of distribution of the included pediatric cases (N=3817) in the review**


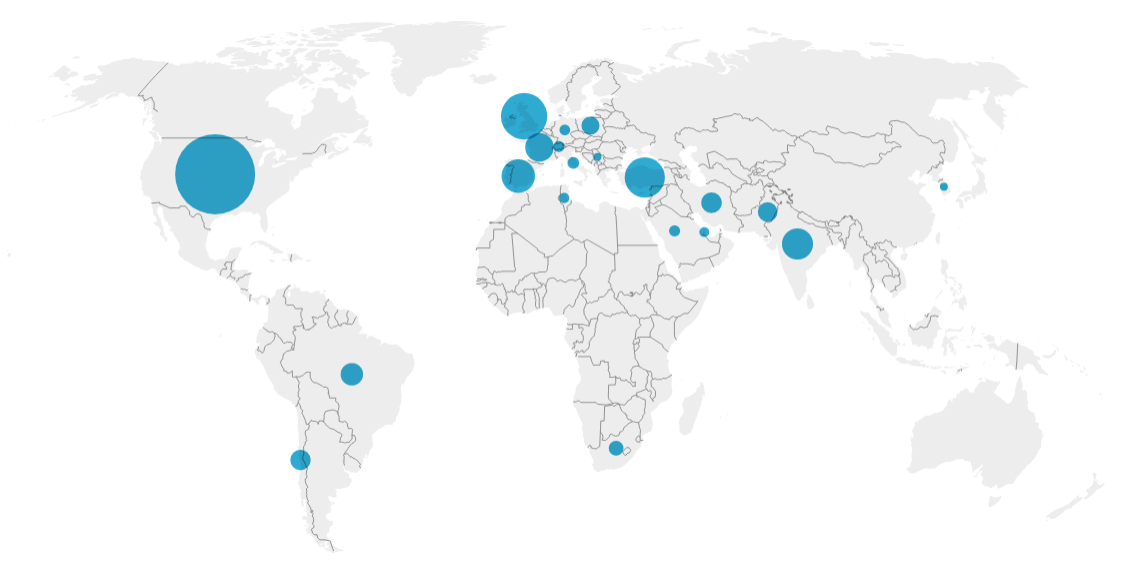


Figure 3. Comparison of Demographics of included HIC vs LMIC pediatric cases in descriptive meta-analysis


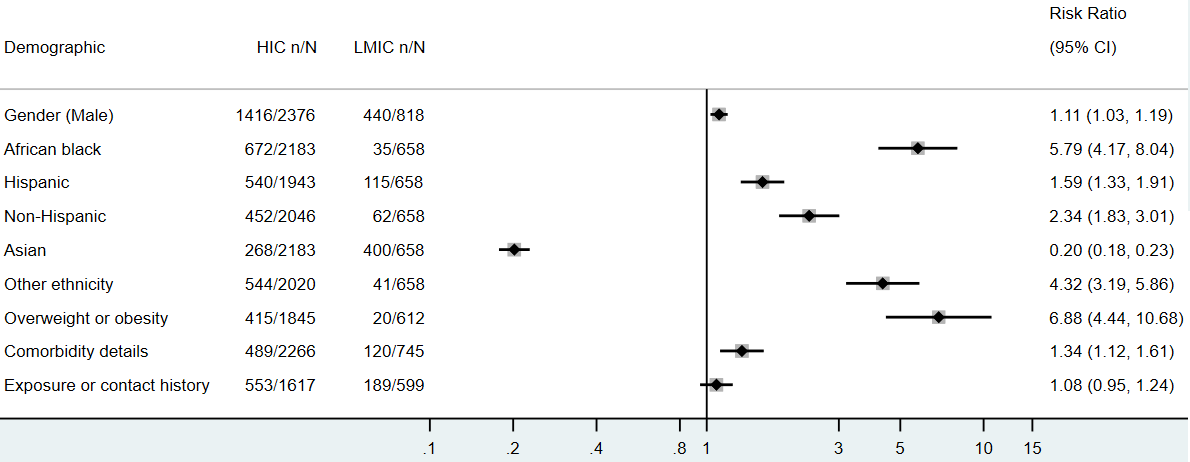


Figure 4. Outcomes of included HIC vs LMIC pediatric cases in descriptive meta-analysis


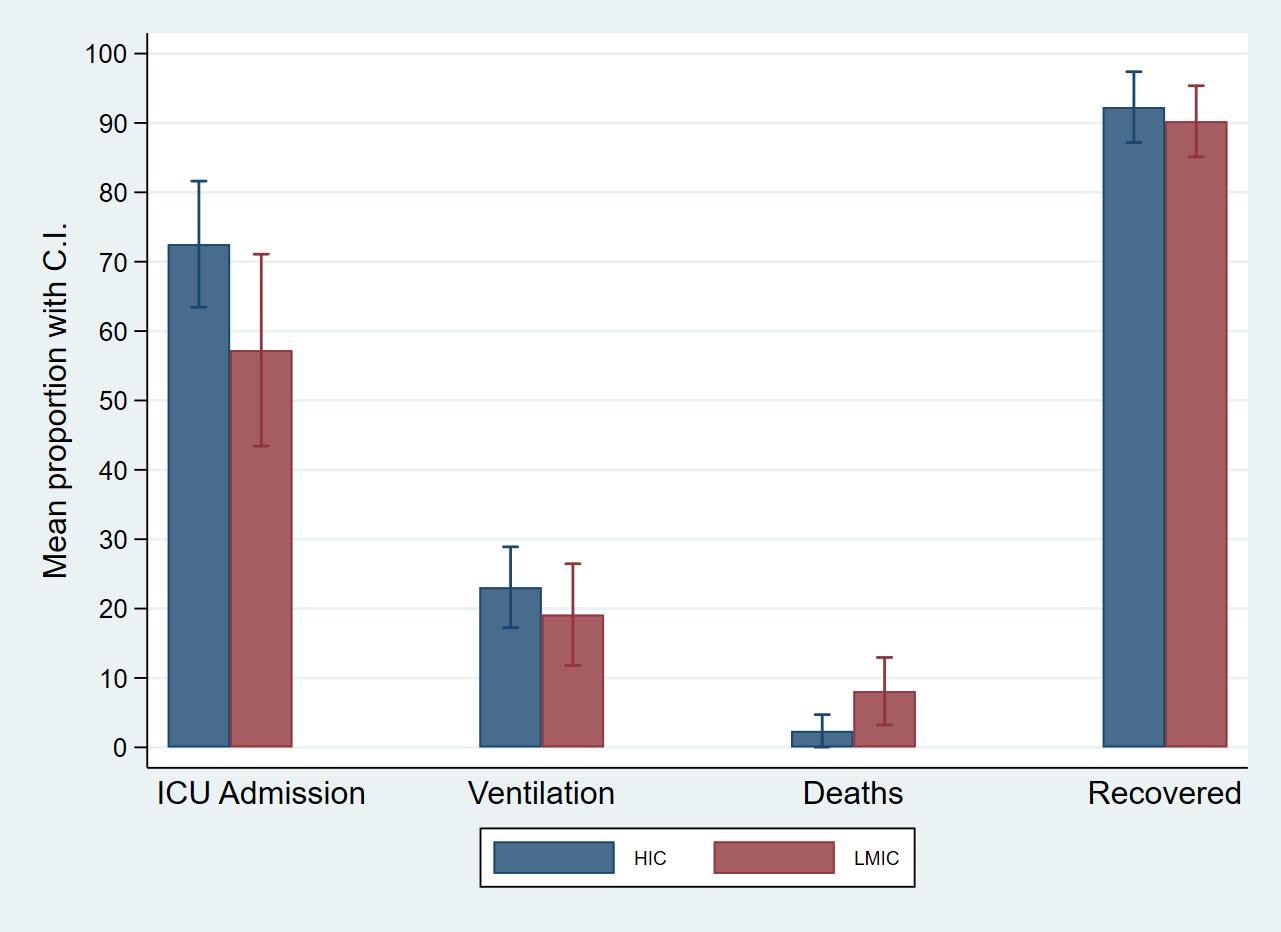

Supplement: Supplementary file 1 — Supplementary file1 (DOCX 524 KB) [file 40124_2022_264_MOESM1_ESM.docx]
